# A Monolithic Janus Hydrogel Pressure Sensor for Wearable Motion and Physiological Monitoring

**DOI:** 10.1002/advs.76893

**Published:** 2026-07-30

**Authors:** Syed Atif Ali, Zeeshan Alam Ansari, Reynaldo Carlos Kuizon Montalbo, Hailemichael Ayalew, Yu‐Sheng Hsiao, Chih‐Wei Chu, Hsiao‐hua Yu, Hsiung‐Lin Tu

**Affiliations:** ^1^ Institute of Chemistry Academia Sinica Taipei Taiwan; ^2^ Department of Physics National Taiwan University Taipei Taiwan; ^3^ Nano‐Science and Technology Program Taiwan International Graduate Program Academia Sinica Taipei Taiwan; ^4^ Research Center for Applied Sciences Academia Sinica Taipei Taiwan; ^5^ Smart Organic Materials Laboratory Institute of Chemistry Academia Sinica Taipei Taiwan; ^6^ Department of Materials Science and Engineering National Taiwan University of Science and Technology Taipei Taiwan

**Keywords:** capacitive pressure sensor, conductive polymer, janus hydrogel, wearable bioelectronics

## Abstract

Sensitive pressure sensors with easy fabrication and stable performance are essential for next‐generation wearable electronics. Here, we report a monolithic Janus hydrogel‐based capacitive pressure sensor composed of two conductive layers and an intervening dielectric layer, forming an all‐hydrogel sensing architecture. The conductive layers are fabricated from poly(3,4‐ethylenedioxythiophene) (PEDOT) incorporated poly(ethylene glycol) diacrylate (PEGDA) hydrogels, while the dielectric layer consists of a pristine PEGDA matrix, enabling intimate interfacial bonding without the need for additional adhesives. The mechanical properties of the hydrogel system reveal tunable swelling behavior and elastic modulus as a function of PEGDA concentration. The resulting sensor displays  a sensitivity of 0.27 kPa^−1^ in the pressure range of 0.49 to 3.43 kPa, and the operating range could extend upto 5.39 kPa. Additionally, the sensor exhibits stable electromechanical performance, featuring a short response time of 23 ms for the rising edge and 36 ms for the falling edge, allowing it to detect the pressure applied under repeated stimuli. Owing to its flexible nature, the designed sensor demonstrates reliable detection of a wide range of mechanical stimuli, from small physiological signals to human motions. Our results highlight that the Janus hydrogel with integrated conductive and dielectric layers could be a promising platform for wearable health monitoring.

## Graphical abstract

This work reports a monolithic Janus hydrogel pressure sensor featuring integrated conductive PEDOT and insulating PEGDA layers. The fabricated device achieves a rapid response time, a wide operating range, and good cyclic durability. The sensor demonstrates robust performance in tracking dynamic human joint motions, offering a scalable strategy for wearable biomonitoring.

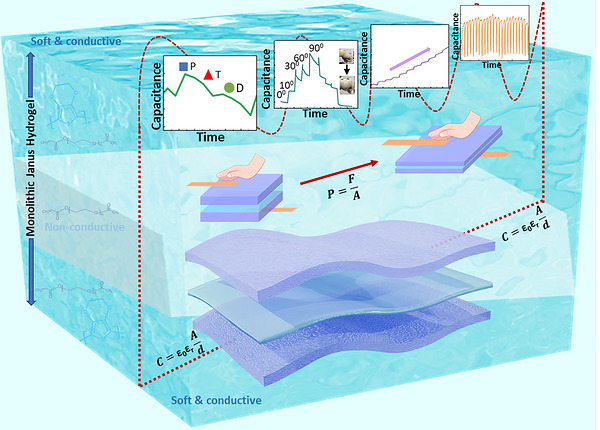

## 1 Introduction

Driven by rapid technological advancements, significant research interest has transitioned toward emerging frontiers in personalized healthcare monitoring, wearable bioelectronics, implantable diagnostics and biomimetic electronic skins. To meet the increasing demands for structural flexibility, light weight integration and easy fabrication, new wearable electronics have been widely explored [1, 2, 3]. By converting traditional rigid electronic components such as resistors, capacitors, and transistors into stretchable and bendable systems through advanced materials and fabrication strategies, flexible electronics enable the integration of complex functions into deformable platforms suitable for next‐generation biomedical applications [4, 5].

Wearable sensors rely on the ability to sense and respond to mechanical deformation. In particular, flexible pressure sensors can detect skin deformation to monitor vital physiological signals, such as pulse rate, respiration, and body motion [6]. These capabilities are important for health management, rehabilitation, and sports training. Moreover, the detection of external stress, joint motion, and actuator deformation facilitates improved Human‐Machine Interface (HMI) and enhances the control of soft robotic systems, thereby contributing to the development of intelligent devices designed to assist human activities [7, 8]. In such applications, pressure variations across different regions of the body provide valuable physiological and biomechanical information, highlighting the importance of flexible pressure sensing technologies.

Pressure sensors can be broadly classified into four categories based on their signal transduction mechanisms: piezoresistive, piezoelectric, capacitive, and triboelectric sensors [9]. Among these, capacitive pressure sensors have attracted significant interest due to their high sensitivity, rapid response, and excellent stability [10, 11, 12, 13]. The performance of these sensors depends on their material selection and structural design. To optimize sensing capabilities, various materials such as graphene [14], carbon nanotubes [15], carbon nanofibers [16], MXene [17, 18], metal nanowires [19], and nanoparticles (NPs) [20] have been utilized to construct efficient sensing devices. Meanwhile, flexible substrates such as poly(dimethylsiloxane) (PDMS) [21], polyimide (PI) [22], polyethylene (PE) [23], and polyurethane (PU) [24] have been used to support active materials and served as wearable electrodes through fabrication techniques such as infilling [25], coating [26], and surface modification [27].

The fabrication of wearable sensors is commonly achieved through template‐based methods [28, 29, 30]. Among these techniques, photolithography remains widely used for producing precise and well‐defined silicon molds for high‐performance pressure sensors [31]. However, photolithography typically requires sophisticated equipment and complex processing steps, which limit its scalability and cost‐effectiveness. As a result, there remains a significant challenge in developing flexible sensors featuring high sensitivity and wide detection ranges while being low cost and can be fabricated with ease. To address these limitations while maintaining excellent conformal contact with biological surfaces, hydrogels have emerged as an important material candidate.

Owing to their three‐dimensional polymeric networks and highly tunable mechanical properties such as stretchability, fracture toughness, and viscoelasticity, hydrogels have attracted substantial attention in the development of flexible wearable sensors [32, 33]. Their intrinsic softness and tissue‐like properties make them particularly suitable for conformal contact with biological surfaces. Particularly, ionically conductive hydrogels are especially promising due to their notable electrical conductivity and adjustable mechanical characteristics [34]. Substantial progress has been made in the development of hydrogel‐based strain sensors with high stretchability and sensitivity [35]. For example, Amstad et al. [36] reported 3D‐printing engineering strategy to fabricate carbon black‐functionalized double network granular hydrogels with high sensitivity and toughness. Recent approaches such as 3D printable high‐performance conducting polymer hydrogels have been developed to create seamless, all‐hydrogel bioelectronic interfaces [37]. However, such approaches often involve complex fabrication procedures and primarily focus on tensile strain sensing rather than the pressure sensing performance.

Conventional capacitive hydrogel pressure sensors typically employ a sandwich configuration consisting of a dielectric layer embedded between two conductive layers [38]. The underlying working principle relies on the deformation of the dielectric layer under external pressure, which alters the capacitance and generates a corresponding electrical signal output [39]. Despite their effectiveness, these structures are highly susceptible to severe deformation and mechanical damage; this vulnerability can compromise electromechanical stability and degrade sensitivity over cycles of repeated use [40]. Although recent studies have explored integrated designs such as gradient charge distribution hydrogels featuring multilayered architectures like cation‐rich, dielectric, and anion‐rich layers, challenges regarding interface delamination and long‐term stability remain [41, 42, 43]. Thus, there is a need for simpler and more robust designs that can enhance sensitivity while maintaining structural integrity and ease of fabrication.

In this work, a monolithic Janus hydrogel‐based capacitive pressure sensor that integrates conductive and dielectric functionalities within single continuous macromolecular architecture was fabricated. To overcome the interfacial mismatch and delamination inherent to conventional capacitive sensors, we engineered PEGDA hydrogel matrix. The conductive regions are polymerized via network formation of poly(3,4‐ethylenedioxythiophene) (PEDOT) within the PEGDA scaffold, wherein the pristine PEGDA domain serves as the elastomeric dielectric layer. Unlike traditional sensors relying on discrete assemblies, this monolithic design ensures seamless viscoelastic coupling across functional zones, suppressing mechanical hysteresis and performance degradation under cyclic strain. The resulting Janus hydrogel demonstrates repeatable compressibility, robust cross‐linking density, and highly reproducible electromechanical transduction. The device reliably resolves deformation signals, such as wrist arterial pulses and respiration, alongside macro‐scale biomechanical motions. By resolving the chronic challenges of interfacial failure and fabrication complexity, this PEDOT/PEGDA hydrogel system establishes a robust platform for smart wearable electronics, HMI, and soft robotic systems.

## 2 Results

### 2.1 Sensing Principle of the Janus Hydrogel Pressure Sensor

**FIGURE 1:**
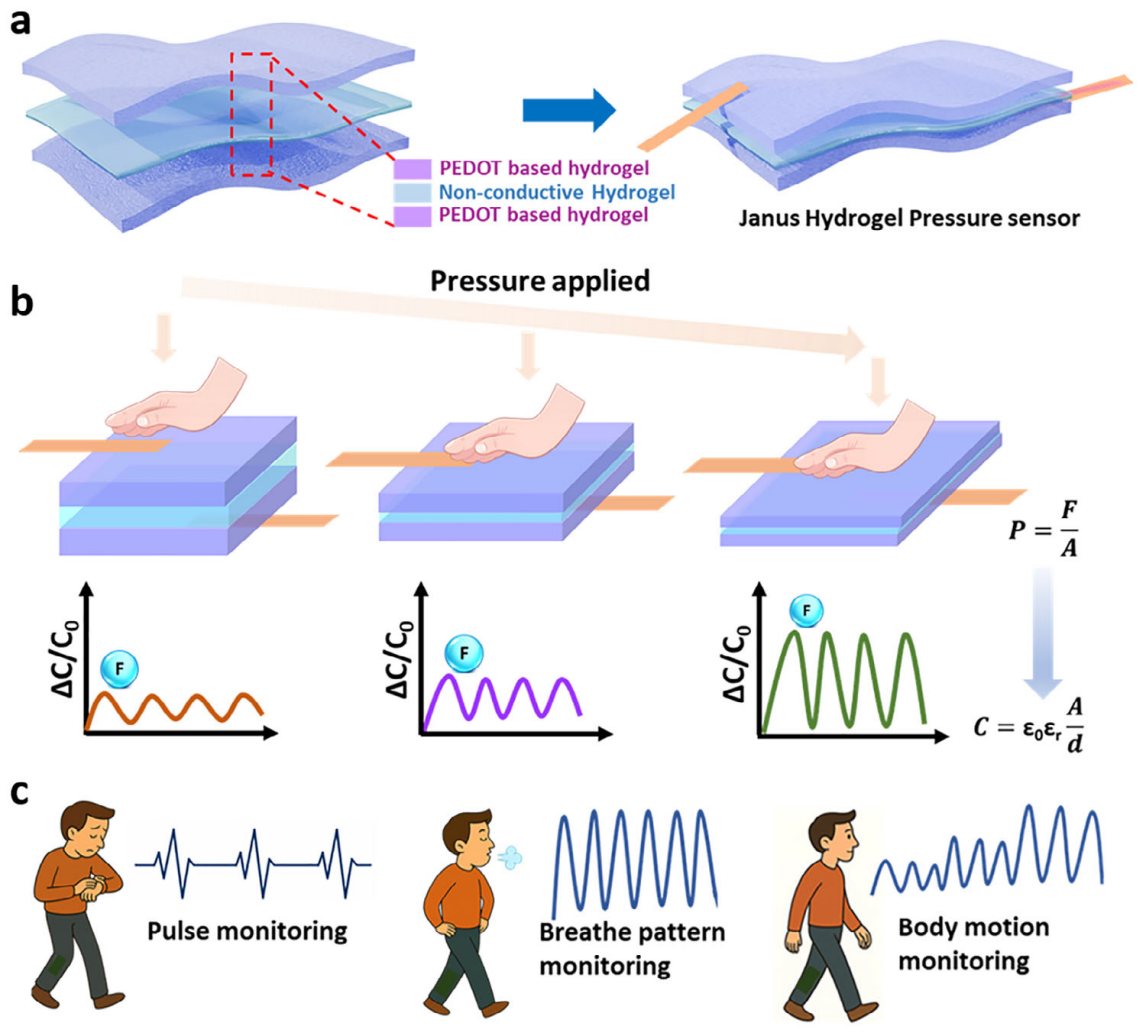
(a) Schematic diagram of a flexible and wearable monolithic Janus hydrogel‐based pressure sensor consisting of PEDOT/PEGDA conductive hydrogel electrodes and PEGDA dielectric hydrogel layer. (b) Assembly of the integrated capacitive pressure sensor with electrical contacts andworking principle of the sensor, showing increased capacitance under increasing applied pressure. (c) Representative images showing applications in physiological and human motion monitoring including arterial pulse, respiration and walking.  . (Diagram created using Chatgpt (accessed March 2026) to generate human schematic images as an initial draft, with subsequent modifications by the authors for Figure c).

The pressure sensor fabricated in this study operates on a capacitive sensing mechanism based on a conductive‐dielectric‐conductive sandwich architecture (Figure 1a,b). The two PEDOT/PEGDA hydrogel layers function as electrodes, while the intermediate PEGDA layer acts as the insulating spacer. Upon application of external pressure, the soft hydrogel structure undergoes elastic deformation, leading to a reduction in the dielectric layer thickness and an increase in the effective contact area between layers. These changes result in a measurable increase in capacitance, which is recorded as the sensor output. Owing to the intrinsic softness and high compressibility of the hydrogel matrix, small pressure variations can be efficiently transduced into changes in electrical signals, enabling sensitive and repeatable pressure detection (Figure 1c).

### 2.2 Preparation and Characterizations of the Electrode Layer and Dielectric Layer

Although numerous studies have reported hydrogel‐based pressure sensors either fabricated via layer‐by‐layer methods [44] or physical stacking components [45], these approaches typically require discrete layered assemblies. To overcome the challenges of requiring rigid polymer networks for efficient charge transfer and interfacial instability inherent to traditional layered assemblies, we optimized electrical conductivity of the system while preserving its intrinsic mechanical stretchability, achieved by selectively engineering an electronically conductive PEDOT network within a cross‐linked PEGDA matrix. Secondly, instead of assembling discrete components through weak physical stacking, the conductive and dielectric layers of this Janus gel was constructed within a single, continuous macromolecular framework.

**FIGURE 2:**
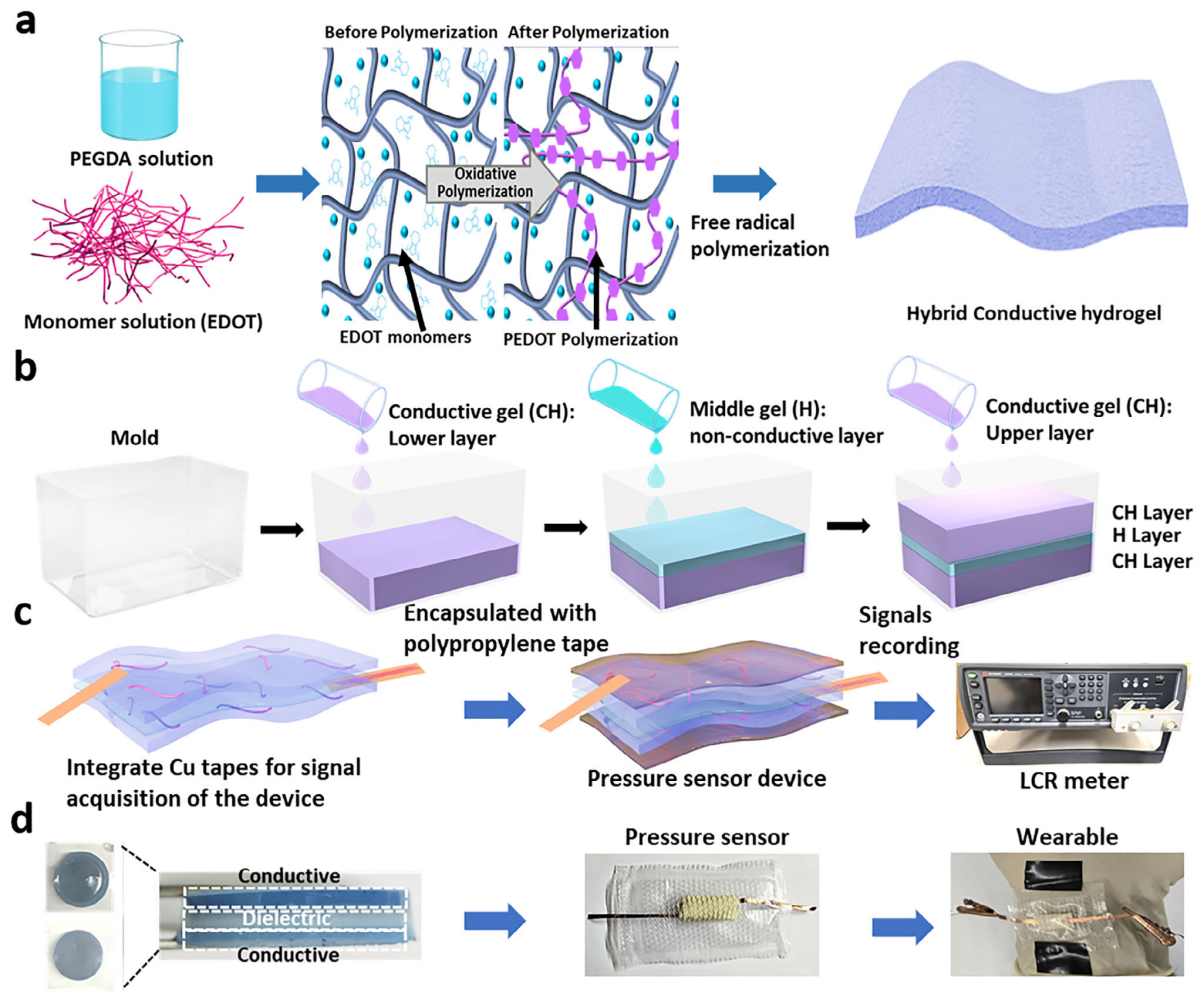
The fabrication process of the monolithic Janus hydrogel pressure sensor. (a) The mechanism of oxidative polymerization of EDOT monomers within the PEGDA matrices. (b,c) The Janus architecture is constructed by sequential casting and polymerization of the bottom conductive layer, the dielectric layer, and the top conductive layer resulting in an integrated three‐layer hydrogel structure with interfacial bonding. (d) Pressure sensor depicting conductive layers, dielectric layer and encapsulated device.

The hydrogel framework was fabricated using a layer‐by‐layer casting approach as given in Figure 2a,b and Figure S1a,b. To optimize the reproducibility and electromechanical performance of the Janus hydrogel pressure sensor, the chemical formulations of the conductive and dielectric layers were optimized. The sensor features a sandwich structure with a total dimension of 15 mm × 10 mm, consisting of two outer conductive layers (each 4.5 mm thickness) and an intermediate dielectric layer (2 mm thickness) (Figure 2).

**FIGURE 3:**
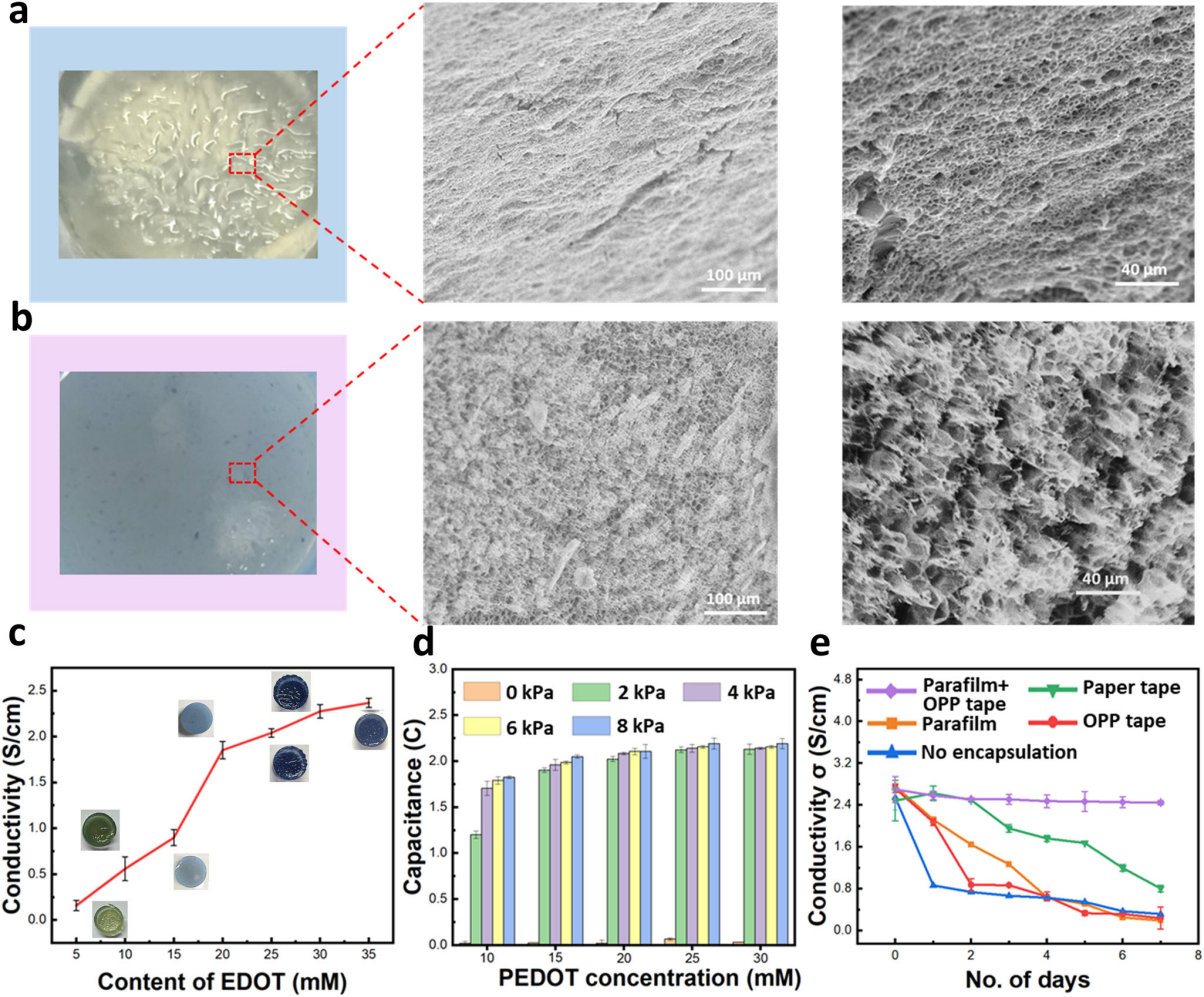
Physical images and Cryo‐scanning electron microscope (SEM) micrographs of (a) PEGDA hydrogel and (b) PEGDA/PEDOT conductive hydrogels. (c) Conductivity of PEGDA/PEDOT layers measured at various EDOT concentrations. (d) Capacitance response of the sensor measured under different applied pressures (0–8 kPa). (e) Long‐term conductivity of the conductive hydrogel under different encapsulation strategies (no encapsulation, OPP tape, paper tape, parafilm, and parafilm + OPP tape).

These hydrogel layers were transformed into a working device by attaching copper tapes on opposite sides (Figure 2c,d). CryoSEM was used to characterize the detailed structure of the non‐conductive and conductive hydrogel (Figure 3a,b). The physical images of the gel also showed color change when PEDOT was polymerized within PEGDA networks. Meanwhile, the SEM images illustrate their surface microstructures before and after preparation of conductive gel, revealing dense fibers of PEDOT polymers over PEGDA hydrogel network. The pure PEGDA hydrogel showed clear pores over the surface, while addition of PEDOT polymers made the pores rougher.

To optimize the electromechanical performance and pressure response of the integrated conductive layers, the EDOT monomer concentration was systematically varied during synthesis. As a baseline material property, the electrical conductivity of the hydrogel was evaluated as a function of EDOT content (Figure 3c). The conductivity exhibited a sharp, progressive rise from approximately 0.15 S/cm to a plateau near 2.4 S/cm as the EDOT concentration increased from 5 to 35 mM, confirming the successful formation of an interconnected, electronically conducting PEDOT network. To ensure robust electrical conductivity while maintaining structural integrity, an optimized final EDOT concentration of 30 mM was selected for the device fabrication.

This enhanced conductivity directly correlates with the sensor's capacitive performance under applied pressure (0–8 kPa). This material testing was conducted on electrode layers to study charge transport dynamics prior to full device integration. At lower EDOT content (15 mm), the conductive network within the PEGDA matrix was insufficiently developed, resulting in limited charge transport and reduced pressure sensitivity. Conversely, utilizing higher concentrations establishes continuous, low‐resistance pathways that facilitate rapid charge distribution and robust electrical double‐layer formation at the internal electrode‐dielectric interface. Beyond a threshold concentration of 20 mM, the gains in both electrical conductivity (Figure 3d) and pressure‐dependent capacitance variation began to saturate, indicating that the charge‐transport pathways had reached maximum percolation. Consequently, an optimized EDOT concentration of 30 mm was selected to balance superior electrical conductivity, high tactile sensitivity, and mechanical integrity for subsequent device fabrication.

The effect of glycerol concentration on the electrical conductivity of the hydrogel was investigated before and after drying (Figure S2a). Prior to drying, the conductivity exhibited a decreasing trend with increasing glycerol concentration, dropping from ∼1.7 S/m at 0% glycerol to ∼0.8 S/m at higher concentrations. This reduction was attributed to the dilution of free ionic species and increased viscosity within the hydrogel network, which limits ion mobility. In contrast, after drying, the conductivity showed an opposite trend, gradually increasing with glycerol content from near‐zero to ∼0.6 S/m at 14% glycerol. This behavior is primarily due to the hygroscopic nature of glycerol, which helps retain moisture within the hydrogel matrix even after drying, thereby preserving ionic pathways and maintaining conductivity. These results highlight the dual role of glycerol in modulating both the initial ionic transport and post‐drying electrical stability of the hydrogel. While higher glycerol concentrations slightly compromise the initial conductivity, they significantly enhance the long‐term electrical performance under dehydrated conditions. Therefore, an intermediate glycerol concentration of 4 wt.% was selected to achieve a balance between high initial conductivity and improved stability after drying, which is critical for reliable operation of the pressure sensor in practical wearable applications.

To evaluate the efficacy of the cross‐linked PEGDA hydrogel as dielectric medium, its frequency‐dependent capacitive response was compared against conventional, dry dielectric layer materials [46] frequently utilized in flexible electronics, specifically polydimethylsiloxane (PDMS) and polypropylene (PP) tape (Figure S2b). The PEGDA hydrogel exhibits a significantly higher capacitance compared to PDMS and PP tape across the entire frequency range (10^2^ to 10^6^ Hz), which presumably was due to its hydrated, ion‐rich network enabling enhanced interfacial and ionic polarization. At low frequencies, the hydrogel shows a sharp increase in capacitance due to the accumulation of mobile ions at the electrode‐electrolyte interface (electrode polarization effect). As the frequency increases, the capacitance gradually decreases and stabilizes, reflecting the limited ability of ions and dipoles to follow the rapidly alternating electric field. In contrast, PDMS and PP tape, being non‐conductive and low‐dielectric polymer materials, display much lower frequency‐independent capacitance values, governed primarily by electronic and dipolar polarization. This comparison highlights the superior dielectric and capacitive response of the hydrogel, making it suitable for sensitive capacitive sensing applications.

The electrical properties of the conductive hydrogel electrode were further evaluated by conductivity study under different encapsulation conditions. To assess the environmental stability of the conductive hydrogel, different encapsulation strategies were compared over time (Figure 3e). The non‐encapsulated sample showed a rapid decline in conductivity, dropping drastically due to dehydration and loss of ionic pathways. Similarly, samples encapsulated with oriented polypropylene (OPP) tape and paper tape exhibited significant conductivity decay, indicating insufficient moisture retention. In contrast, parafilm‐based encapsulation demonstrated improved stability, while the combination of parafilm and OPP tape provided the most effective protection, maintaining conductivity at around 2.4 S/cm over the testing period with minimal degradation. This enhanced stability was marked by reduced water evaporation and preservation of the hydrogel's internal ionic environment. Taken together, these results highlight that both EDOT concentration and encapsulation strategy is crucial in governing the electrical performance and durability of the conductive hydrogel electrode. An optimized EDOT concentration, combined with effective encapsulation (parafilm + OPP tape), was thus selected to ensure high conductivity and long‐term stability for reliable sensor operation.

Figure S3a shows the capacitance variations under changing compressive pressure over a broad frequency range (0 to 10 000 Hz). As demonstrated in the frequency sweeps, the conductive gel exhibits a capacitive increment in the low‐frequency (below 2000 Hz), regardless of the applied load. This characteristic is governed by interfacial polarization (the Maxwell‐Wagner‐Sillars effect), where mobile ions have sufficient time to migrate and accumulate at the internal phase boundaries to form an electrical double layer (EDL). At higher operating frequencies, the electric field switches too rapidly, causing the capacitance to stabilize into a frequency‐independent range. While increasing the applied pressure from 0.075 to 20 kPa shifts the capacitive curve upward across the entire frequency range. This rise confirms that mechanical compression effectively decreases the internal dielectric thickness while simultaneously increasing the ion concentration, establishing a reliable, pressure‐dependent electrical output.

To further support the capacitive mechanisms of the Janus hydrogel sensor, electrochemical impedance spectroscopy (EIS) was performed. The Nyquist plot (Figure S3b) shows a depressed high‐frequency semicircle followed by a low‐frequency inclined tail, indicating a combined contribution from bulk ionic transport and interfacial polarization. The data were fitted using an equivalent circuit consisting of a bulk resistance (*R*_*s*_) in series with a parallel combination of polarization resistance (*R* *_p_*) and a constant phase element (CPE), which shows the non‐ideal capacitive response at the hydrogel‐electrode interface (Table S1). The fitted bulk resistance, *R*_*s*_ = 60.98 Ω, suggests efficient ionic conduction through the hydrated hydrogel network. The higher polarization resistance, *R*_*p*_ = 6181 Ω, indicates that interfacial charge accumulation at the hydrogel‐electrode boundary dominates the low‐frequency response. The CPE exponent, *n* = 0.675, deviates from the ideal capacitive value of *n* = 1, confirming non‐ideal interfacial capacitance. This behavior might be due to structural heterogeneity, surface roughness, and spatially distributed ion migration pathways within the Janus hydrogel. Therefore, the EIS results support the presence of interfacial polarization and EDL‐related charge storage, while the low *R*_*s*_ confirms that bulk ionic transport remains favorable.

### 2.3 Dielectric, Swelling, and Mechanical Characterization of the Hydrogel

**FIGURE 4:**
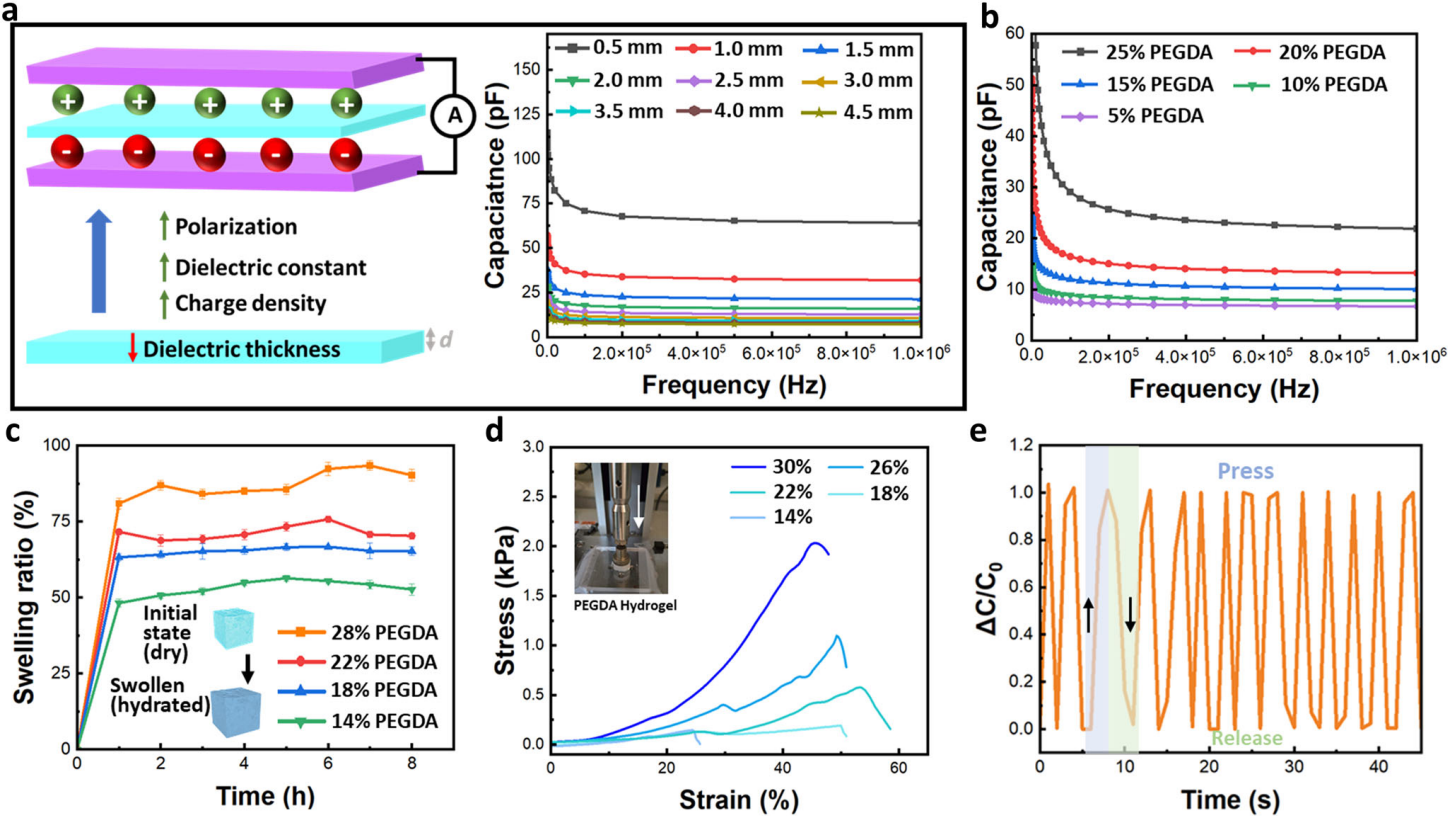
Mechanical characterization and electromechanical response of PEGDA‐based hydrogels. (a) Schematic illustration of the effect of dielectric thickness on the capacitance. (b) Capacitance of PEGDA hydrogels with different polymer concentrations (5%–25%) measured over a frequency range of 10^2^–10^6^ Hz. (c) Time‐dependent swelling ratio of PEGDA hydrogels with different polymer concentrations (14%–28%), (d) Compressive stress‐strain curves of the PEGDA hydrogels at varying polymer concentrations (inset: photograph of the experimental compression setup). (e) Real‐time normalized capacitance change (ΔC/C_0_) of the pressure sensor under repeated pressing and releasing cycles, showing fast response, good reversibility, and stable signal output during dynamic mechanical loading.

To elucidate the influence of material composition, geometric thickness, and operational frequency on the capacitive behavior of the sensor, its dielectric properties were systematically investigated as illustrated in Figure 4. The working mechanism (Figure 4a) demonstrates that the capacitance is inversely proportional to the dielectric thickness; under applied pressure, the reduction in dielectric thickness leads to an increase in capacitance, forming the basis of pressure sensing is expressed by Equation (1).

(1) $$C=\frac{\varepsilon_{0}\varepsilon_{r}A}{d}$$

where *ε_0_* is the vacuum permittivity, *ε_r_* is the relative dielectric constant of the hydrogel, *A* is the effective electrode area and *d* is the dielectric layer thickness.

The effect of dielectric layer thickness on capacitance was further examined. As expected, thinner dielectric layers resulted in higher capacitance values across the entire frequency range, consistent with the fundamental capacitance relation. Increasing thickness from 0.5 to 4.5 mm led to a systematic decrease in capacitance, confirming that dielectric thickness is a critical parameter for tuning sensor sensitivity. Thinner layers enhance sensitivity but may compromise mechanical robustness, indicating the need for an optimized balance. Next, the dielectric response of PEGDA hydrogels with varying polymer concentrations (5%–25%) was evaluated over a frequency range of 10^2^–10^6^ Hz (Figure 4b). A clear trend of decreasing capacitance with increasing frequency was observed for all compositions, which can be attributed to the reduced ability of dipoles and ions to follow high‐frequency electric fields. Reduced ionic mobility causes capacitance decreases because ions cannot migrate efficiently to the electrodes to form the electric double layer (EDL). Higher capacitance in denser PEGDA networks is driven by increased dipolar density and interfacial polarization (Maxwell–Wagner–Sillars effect) at the internal boundaries [47]. The swelling behavior of PEGDA hydrogels with different polymer concentrations was calculated according to the Equation (2).

(2) $$SR\left(\%\right)=\frac{W_{s}-W_{d}}{W_{d}}\times 100$$

where *SR* is swelling ratio, *W_s_* is swollen weight and *W_d_* is dry weight of the gel. The swelling ratio was evaluated over time, as shown in Figure 4c. For each PEGDA concentration, three hydrogel replicates (*n* = 3) were tested simultaneously to calculate the average equilibrium swelling ratio. All samples exhibited a rapid increase in swelling ratio within the first hour, indicating fast water uptake due to the hydrophilic nature of the polymer network. Following the initial phase, the swelling gradually approached equilibrium, with only minor changes observed after 4–6 h. Notably, a clear dependence on PEGDA concentration was observed. Hydrogels with higher polymer content (28%) showed the highest swelling ratio (90%–95%), while lower concentrations (14%) exhibited significantly reduced swelling (50%–55%). PEGDA is highly hydrophilic due to the repeating ethylene oxide units in its backbone. At a higher polymer concentration of 28 wt.%, greater volumetric density of these water‐attracting ether groups is present compared to 14 wt.%, creating a stronger thermodynamic driving force (osmotic pressure) to draw water into the gel. In contrast, lower concentration hydrogels form looser networks with reduced water retention capacity. The swelling behavior directly influences the dielectric and mechanical properties of the hydrogel. Higher swelling ratios contribute to increased ionic mobility and dielectric constant, which are beneficial for capacitive sensing performance. However, excessive swelling may compromise mechanical stability. When the polymer precursor concentration is increased further to high content, the equilibrium swelling ratio may begin to decline substantially. This is due to the lower degree of crosslinking at the lower monomer limits (14 wt% and 18 wt%). Therefore, an intermediate PEGDA concentration was selected to balance water retention, mechanical integrity, and electrical performance for optimal sensor functionality. The mechanical properties of the samples were evaluated by comparing their strain vs stress curve using compression testing as a function of precursor concentration. The compression measurements were conducted at a constant testing speed (T‐Speed) of 6 mm/min, ensuring a uniform loading rate across all hydrogel samples to eliminate time‐dependent relaxation variations. While to maintain consistency with standard hydrogel characterization protocols, stress was reported based on the pristine initial cross‐sectional area (*A_0_*), which represents a baseline for wearable sensor devices. As shown in Figure 4d, all samples display characteristic viscoelastic behavior with a distinct strain‐hardening profile under increasing load. A systematic increase in PEGDA content from 14% to 30% yields an increase in the maximum compressive stress from ∼0.1 to 2.0 kPa. This tunable mechanical property is directly governed by the higher polymer crosslinking density, which restricts crosslinking networks and enhances the structural network stiffness required for durable wearable sensor applications.

Overall, the results demonstrate that the mechanical properties can be tailored to balance flexibility and structural stability for optimized sensor performance. While the 30 wt% PEGDA hydrogel displays the highest compressive strength (∼2.0 kPa), its dense network limits pressing property under low‐pressure loads. On the other hand, the 14 and 18 wt.% gels are better for wearable devices but exhibit poor structural integrity for structural deformation under cyclic testing. Therefore, the 22 and 26 wt.% PEGDA was selected as the optimized matrix for the wearable sensor fabrication. To evaluate the relationship between network architecture and bulk mechanical properties, the Young's modulus *(E)* was plotted vs monomer weight (Figure S4a). Young's Modulus *(E)* was calculated using the Equation (3).

(3) $$E=\frac{\Delta \sigma }{\Delta \varepsilon }$$

where *Δσ* is change in stress and *Δε* is change in strain. The modulus exhibits a non‐linear behavior, increasing from less than 0.1 kPa at 14% PEGDA to ∼1.22 kPa at 30% PEGDA. This upward trend confirms that increasing the monomer concentration increase the cross‐linking density of the polymer network.

The dynamic response of the sensor under cyclic mechanical deformation was evaluated to assess its stability and repeatability (Figure 4e). The device was subjected to periodic pressing/bending and releasing cycles, resulting in a consistent and repeatable signal output over time. The ΔC/C_0_ signal exhibited a uniform, triangular waveform corresponding to the applied strain cycles, with clear distinction between the loading (pressing/bending) and unloading (releasing) phases. A stress relaxation test was conducted on the hydrogel matrix using the rheometer. The hydrogel was subjected to an instantaneous compressive strain and held at a constant pressure over a period while the normal force response was continuously monitored. As shown in Figure S4b, the hydrogel exhibits viscoelastic relaxation behavior. Upon reaching the target strain, an immediate decay in stress is observed, followed by a steady‐state equilibrium value.

The interfacial integrity of the monolithic Janus hydrogel was evaluated by fluorescence imaging using FITC‐dextran as a tracer dye (Figure S5). FITC‐dextran was incorporated only into the middle PEGDA dielectric layer prior to polymerization, enabling selective visualization of the middle layer architecture and interfacial integrity. To evaluate the interfacial strength of the hydrogel, sliding tests were conducted on both the optimized monolithic architecture and sequential layered control sample. The intact device exhibited a continuous and well‐defined fluorescent middle layer with no visible peeling, confirming successful monolithic integration (Figure S5a). The strong interfacial adhesion between successive hydrogel layers originates from the free‐radical copolymerization that occurs during the sequential layer‐by‐layer fabrication process. After casting the first PEGDA layer, polymerization is initiated by the APS/TEMED redox system. Because free‐radical polymerization does not consume all acrylate groups, residual acrylate double bonds and active radical chain ends remain at the surface during the early stages of gelation [48, 49]. When the precursor solution of the subsequent layer is cast before complete network formation, PEGDA monomers diffuse into the swollen interfacial region of the underlying hydrogel. Upon initiation of polymerization in the newly added layer, these diffused monomers copolymerize with the residual surface acrylate groups through covalent bonding, while simultaneous chain propagation across the interface creates an interpenetrating polymer network (IPN) (Figure S1). The combination of covalent interfacial crosslinking and polymer‐chain entanglement eliminates a distinct weak boundary between adjacent layers, resulting in a mechanically integrated monolithic hydrogel that resists delamination under bending and peeling.

In contrast, a control sample was prepared by allowing the lower hydrogel layer to fully polymerize, followed by casting the middle PEGDA precursor solution onto its surface after a 10 min interval. This sample exhibited obvious interfacial separation during peeling, indicating weak adhesion between independently formed layers (Figure S5b). The layers were held together primarily by weak physical interactions, leading to interfacial separation during peeling. These results demonstrate that sequential polymerization through the monolithic fabrication strategy produces strong interlayer bonding, effectively eliminating delamination while maintaining structural integrity under mechanical deformation.

The electromechanical performance of the hydrogel‐based capacitive sensor was evaluated under different loading conditions, as illustrated in Figure S6. Upon application of a constant pressure, the sensor exhibits a rapid increase in the normalized capacitance change (ΔC/C_0_), followed by a gradual relaxation over time (Figure S6a–c), which can be attributed to the viscoelastic nature of the hydrogel and the redistribution of internal ionic species. These studies show a delayed but sharp rise in ΔC/C_0_, indicating time‐dependent mechanical deformation and charge stabilization under sustained loading. Moreover, the sensor demonstrates a clear and stepwise increase in capacitance with incrementally applied pressures (Figure S6d), confirming its capability to distinguish multiple pressure levels with good resolution and repeatability. The progressive increase in ΔC/C_0_ with pressure is due to the reduction in dielectric thickness and enhanced interfacial polarization within the hydrogel matrix. Additionally, the observed relaxation behavior highlights the coupled electromechanical response of the system, where mechanical deformation and ionic migration collectively influence the capacitance signal. These results demonstrate that the sensor possesses high sensitivity, stable response, and reliable performance for dynamic and static pressure sensing applications.

### 2.4 Device Response Characterization Under Dynamic Force & Stimuli

**FIGURE 5:**
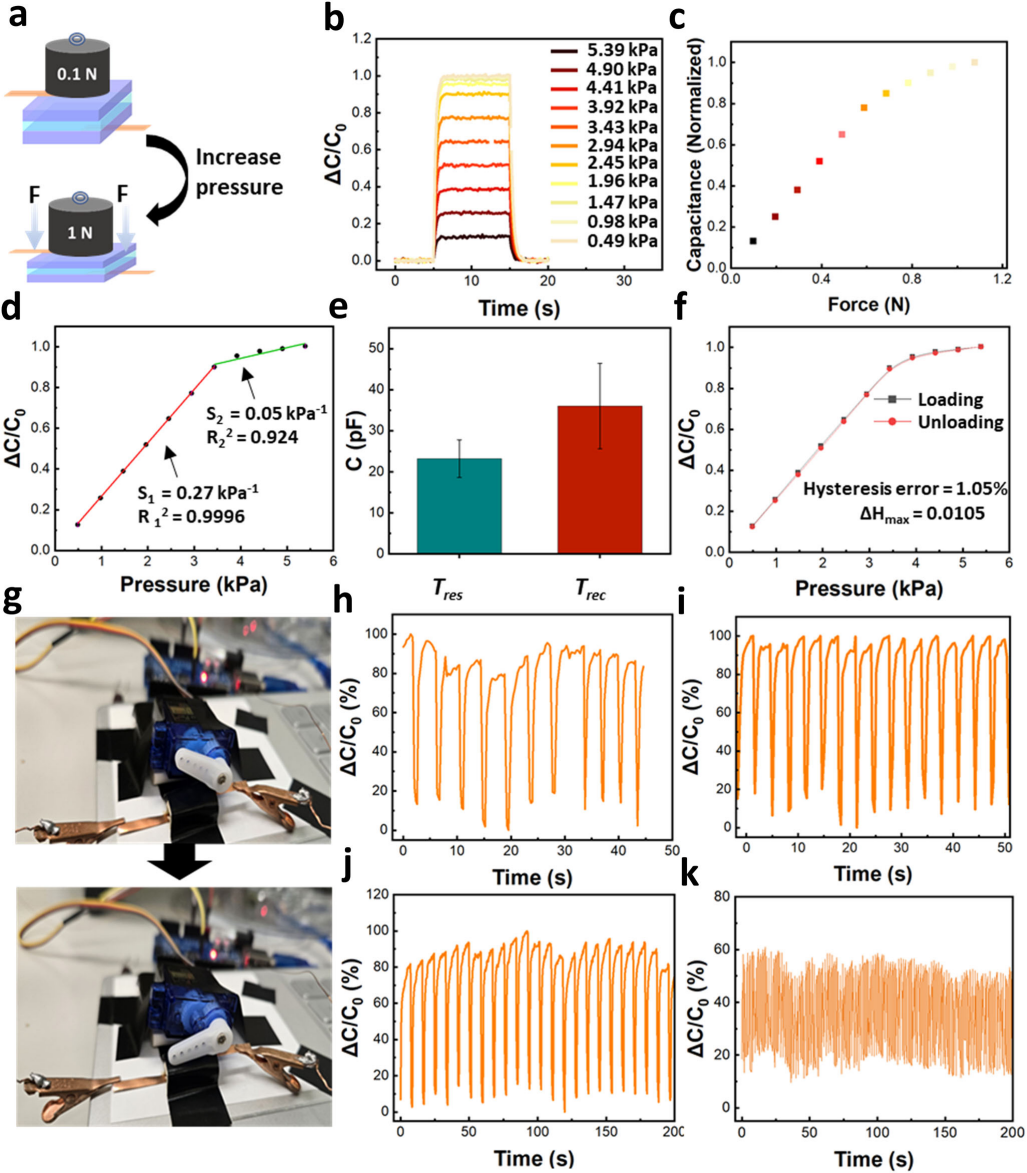
(a) Pressure sensing performance of the sensor under different applied weights. (b,c) Effect of various pressure ranges on sensor. (d) Relative capacitance of pressure sensor exhibiting a bi‐linear response. (e) Graph depicting response and recovery states with loading and unloading of weights. (f) Hysteresis characteristics evaluated across a loading‐unloading of pressure. (g) Rotary actuator pressing pressure sensor at different speed controlled using Arduino. (h–k) Response of sensor using pressing of rotator at different speeds from slow to fast.

The dynamic performance of the fully integrated pressure sensor was further evaluated under different forces and motion responses, as presented in Figure 5. The force input *(F)* was converted to applied pressure *(P)* based on the active device loading area (*A*:15 mm x 10 mm = 150 mm^2^) using the relation, *P = F/A*. Based on the optimization values in Figure 3d, the operational limit of the Janus gel sensor approaches saturation near ∼5 kPa. Therefore, subsequent dynamic electromechanical evaluations (Figure 5a) were conducted within the working range of 0 to ∼6 kPa to accurately find out the device's sensitivity performance. Figure 5a–c illustrate the sensor response to varying force levels, where distinct and stable ΔC/C_0_ signals were obtained for pressures ranging from 0.49 to 5.39  kPa. The capacitance increased systematically with applied force, and the normalized response showed a nearly linear trend with increasing force, confirming the sensor's good sensitivity and reliable pressure discrimination over the tested range. The limit of detection (LOD) was found to be 0.49 kPa, which serves as the lower value for the operational window of 0.49–3.43 kPa. Sensitivity, S for capacitive pressure sensors was calculated using the Equation (4).

(4) $$S=\frac{\Delta C/C_{0}}{\Delta P}$$

ΔC/C_0_ means normalized capacitance change, P is applied pressure. The resulting calibration curve exhibits a bilinear response, indicating two distinct operating pressure ranges governed by the viscoelastic deformation behavior of the soft PEGDA dielectric layer. In the low‐pressure (0.49 to 3.43 kPa), the sensor demonstrates sensitivity (S_1_) of 0.27 kPa^−1^ with good linearity (R_1_^2^ = 0.9996; y = 0.263x + 0.0009). As the pressure increases into the secondary pressure range (3.43 to 5.39 kPa), the sensor undergoes structural changes where the polymer network approaches its mechanical limits. Consequently, the curve transitions to a shallower slope with a sensitivity (S_2_) of 0.05 kPa^−1^ (R_2_^2^ = 0.9238; y = 0.0315x + 0.8357) (Figure 5d).

After 4 kPa, the curve begins approaching saturation, so sensitivity decreases reflecting the typical nonlinear response at higher loads. The dynamic response characteristics of the pressure sensor were evaluated under cyclic loading‐unloading conditions, as shown in Figure S7. The sensor exhibits a stepwise increase in normalized capacitance (ΔC/C_0_) with increasing applied pressure, followed by a symmetric decrease during unloading, indicating good reversibility.

To evaluate the responsiveness of the sensor, the response time (*T_res_*) was calculated using *T_res_ = t_2 –_ t_1_*, where t_1_ and t_2_ represent the specific times at which the relative capacitance change reaches 10% and 90% of its maximum steady‐state value, respectively, upon a sudden step applied pressure. Similarly, the recovery time (*T_rec_*) was calculated using *T_rec_ = t_4 –_ t_3_*, where t_3_ and t_4_ denote the specific times at which the relative capacitance change reaches 10% and 90% of its maximum steady‐state value, following sudden pressure unloading. As highlighted in the inset figures, the sensor demonstrates a fast response time of 23.0±4.6 ms (mean±SD, *n* = 5) upon loading and a recovery time of 36±10.4 ms (mean±SD, *n* = 5) upon unloading (Figure 5e). The rapid signal transition reflects efficient electromechanical coupling within the hydrogel matrix, arising from instantaneous structural deformation and recovery combined with fast ionic redistribution.

To compare the performance of the engineered pressure sensor, its response was evaluated against recently reported state‐of‐the‐art flexible capacitive and polymer‐based pressure sensors (Table S2). The observed response time of 23 ms outperforms some conventional ionic hydrogel‐based sensors, which typically exhibit response times ranging from 50 ms to over 150 ms due to inherent viscoelastic relaxation and bulk ionic transport [50, 51]. Furthermore, the sensitivity achieved in this study is comparable to those achieved by advanced microstructured ionic gel matrices, such as the sandpaper‐templated PVA/carbon nanoparticle networks [52] and multi‐crosslinked dual‐network ionic hydrogels [44]. While specialized architectures utilizing fast‐response structures such as the nylon netting sandwich configuration (<20 ms) or multi‐crosslinked PVA/chitosan networks (11 ms) exhibit marginally faster response, while they are limited by structural fabrication constraints. Hence, the consistent and repeatable signal evolution observed for our sensor over multiple steps confirms the reliability and stability of the sensor under dynamic operations, making it suitable for real‐time pressure monitoring applications.

To further evaluate the electromechanical reliability and viscoelastic recovery of the sensor, the degree of hysteresis (*H*) was systematically quantified across the working range (Figure 5f). The hysteresis error was calculated according to Equation (5).

(5) $$H\left(\%\right)=\frac{{\left|\left(\Delta C/C_{0}\right)\mathrm{loading}-\left(\Delta C/C_{0}\right)\mathrm{unloading}\right|}_{\max}}{\left(\Delta C/C_{0}\right)\max}\;x\;100$$

where the numerator denotes the maximum absolute discrepancy (*ΔH_max_*) between the consecutive loading (compression) and unloading (decompression) capacitance pathways at an identical pressure baseline, and (*ΔC/C_0_*)_max_ represents the maximum capacitive response. As illustrated in Figure 5f, the loading and unloading curves nearly overlay throughout the entire pressure range, demonstrating less hysteresis error of just 1.05% with a maximum signal gap (*ΔH_max_*) of 0.0105 at an applied pressure of 1.47 kPa. These results indicated better elastic recovery and structural stability of the monolithic hydrogel sensor.

The dynamic performance and mechanical tracking reliability of the pressure sensor was evaluated using a rotary actuator (servo motor) controlled via an Arduino platform (Figure 5g and Video S1). The comprehensive electrical circuit and hardware wiring diagram for the microcontroller‐servo integration are provided in Figure S8. The actuator was programmed to apply periodic pressure on the sensor at different speeds, enabling controlled and repeatable mechanical stimulation. The sensor exhibited clear and periodic ΔC/C_0_ responses corresponding to the cyclic pressing‐releasing process (Figure 5h–k). At lower actuation speeds, well‐defined peaks with longer intervals are observed, indicating enough time for complete deformation and recovery of the hydrogel matrix. As the pressing speed increases, the frequency of the peaks increases accordingly, while the amplitude remains relatively stable, demonstrating the sensor's ability to track rapid mechanical inputs without significant signal degradation. At the highest speeds, a more continuous and denser signal pattern is observed due to reduced relaxation time between successive cycles. These results confirm the rapid dynamic response, fast recovery behavior, and frequency‐dependent sensitivity of the sensor, highlighting its suitability for real‐time monitoring of both slow and fast human motions.

### 2.5 Applications of Pressure Sensor for Physical Body Movements

**FIGURE 6:**
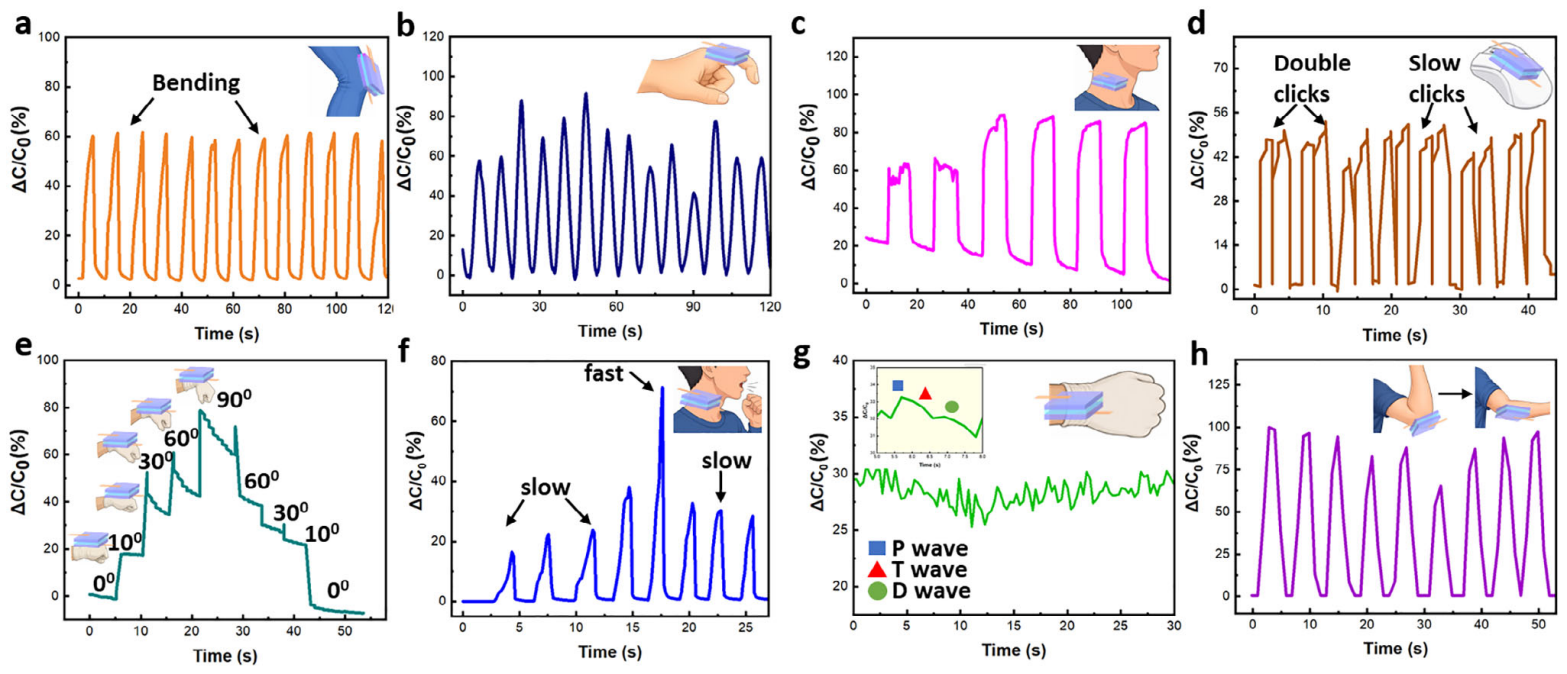
Demonstration of wearable sensing applications using the monolithic Janus hydrogel pressure sensor. Stepwise change in normalized capacitance (ΔC/C_0_) in response to sequential pressing, indicating detection of different pressure levels. (a) Knee bending response, (b) sensor response during finger bending, (c) the output response of the neck movement, (d) response of the pressure sensor for mouse clicks, (e) wrist bending response at different angles, (f) signal response during cough, (g) Real‐time wrist pulse monitoring using the sensor attached to the radial artery. Characteristic features corresponding to the percussion (P), tidal (T), and dicrotic and (h) elbow bending response. (Diagram created using Chatgpt (accessed May 2026) to generate partial human schematic images as an initial draft, with subsequent modifications by the authors for Figure a,b,c,e,f,g,h).

The practical applicability of the sensor for wearable sensing was demonstrated through monitoring of various human physiological signals and body motions, (Figure 6). The sensor exhibited a clear stepwise increase in normalized capacitance (ΔC/C_0_) in response to sequential pressing, indicating its ability to reliably distinguish different pressure levels with stable signal output and minimal fluctuation.

The sensor also showed reliable performance in detecting dynamic body movements. In knee joint motion (Figure 6a), the sensor generated stable and consistent signals under large deformation, highlighting its capability to monitor high‐amplitude movements without signal degradation. Similarly, during finger bending (Figure 6b and Figure S9a), repeatable and reversible capacitance changes were observed over multiple bending‐releasing cycles, indicating good flexibility and durability. Notably, slight variations in peak intensities were observed across individual cycles. This variation is attributed to minor fluctuations in the manual finger‐bending angles and deformation speeds during real‐time human testing. Furthermore, the real‐time sensing capability was demonstrated by monitoring dynamic neck movements (Figure 6c). The sensor displayed distinct and repeatable signals corresponding to periodic physiological motions, such as muscle activity. Each cycle produced a sharp increase in ΔC/C_0_ followed by a rapid recovery to baseline, indicating fast response and recovery behavior. The variation in peak amplitude suggests differences in the intensity or nature of the applied mechanical stimulus. The real‐time response curve displays two distinct lower‐amplitude peaks during the first 40 s, corresponding to initial partial finger‐bending actions. Subsequently, the sharp rise in peak amplitudes to a stable peak (∼85), corresponding to full‐amplitude, deep finger bending, and demonstrating the sensor's ability to clearly differentiate between different degrees of joint movement.

The practical utility of the sensor for subtle physiological monitoring was also confirmed by testing human respiration patterns across multiple days (Figure S9b). The recorded signals maintained consistent periodic patterns, although slight variations in amplitude and frequency were observed, likely due to natural physiological differences. The sensor can differentiate between deep breathing characterized by higher capacitive amplitudes and normal breathing on Day 1, Day 3, and Day 5, demonstrating stable baseline stability and long‐term operational repeatability for wearable healthcare diagnostics. To monitor clicking, a pressure sensor was employed to recognize the click frequency of a mouse through real‐time monitoring (Figure 6d). Double clicks (2 times in 3 s) and slow clicks (1 time in 1 s) were applied on the left mouse button, respectively, triggering a slow signal response. The degree of the applied signal to the output signal indicates that the pressure sensor has promising recognition performance for click speed and may be utilized in HIMs for health monitoring.

As in Figure 6e and Figure S10a, recognition of the bending ability and bending degree of joints was accomplished using wrist and finger movement, respectively. These metrics are critical for movement count and angle analysis and for applications involving motion monitoring and rehabilitation training of patients with arthritis. Figure 6f presents the response signal curve of a sensor on a throat when the subject coughed once slowly, once rapidly, and again slowly in sequence. The sensor produced responses for different degrees of cough, showcasing our sensor design's application in disease diagnosis, such as asthma and influenza. For physiological monitoring, the sensor was attached to the wrist to detect radial artery pulse (Figure 6g and Figure S10b). Distinct waveform features corresponding to the percussion (P), tidal (T), and dicrotic (D) peaks were resolved, demonstrating the high sensitivity and fast response of the device in capturing small biomechanical inputs. This confirms the suitability and potential of the sensor for real‐time cardiovascular monitoring. As demonstrated in Figure 6h, the sensor was attached to the elbow to monitor elbow joint movements in real time.. The initial cycles (0–15 s) yield maximum capacitance changes, corresponding to complete elbow bending. As the degree of bending is reduced during intermediate cycles (15–35 s), the amplitudes exhibit a stepwise decline, reaching a minimum of ∼65. The immediate recovery of peak heights upon returning to full bending (35–52 s) highlights the sensor's precision and rapid responsiveness to diverse biomechanical ranges of motion. To evaluate device‐to‐device reproducibility during wearable applications, a batch of three independent sensors (*n* = 3) was evaluated. The results show low relative standard deviation (RSD of 1.21%–4.47%) (Figure S10c) across three different motions. This uniformity confirms the structural and electromechanical predictability of the developed sensor.

### 2.6 Stability Testing

The short‐term cycling stability and durability of the pressure sensor were evaluated under continuous cyclic loading, as illustrated in Figure S11a. The results represent normalized capacitance change (ΔC/C_0_) recorded over 3000 cycles during repeated pressing‐releasing cycles. The sensor exhibited stable and periodic signals throughout the entire testing duration, with only negligible decrement in amplitude. This consistent response indicates mechanical stability and stable electromechanical signals within the hydrogel structure, even under prolonged operation. To further examine signal stability, enlarged views of the initial and final stages were analyzed. During the first 100 s, the sensor displayed uniform peak amplitudes and well‐defined waveform shapes, confirming reliable initial performance. The minimal variation between the initial and final stages demonstrates the sensor's strong resistance to mechanical fatigue and structural degradation. Furthermore, the sensor's long‐term repeatability was validated over continuous cycles. The relative standard deviation (RSD) of the peak yielding a low variation of 2.16%. This negligible baseline drift confirms that the crosslinked interpenetrating network effectively mitigates viscoelastic fatigue under repetitive mechanical deformation. This mechanical stability is consistent with recent study on ion‐mediated strategy aimed at repairing and strengthening complex covalently cross‐linked double‐network hydrogels [53]. Cross‐sectional analysis was conducted using optical microscopy to evaluate the interfacial integrity of the Janus hydrogel matrix. As shown in Figure S11b, the interface between the distinct conductive and dielectric layers remains continuous and intact with no visible delamination after cyclic pressing operations.

**FIGURE 7:**
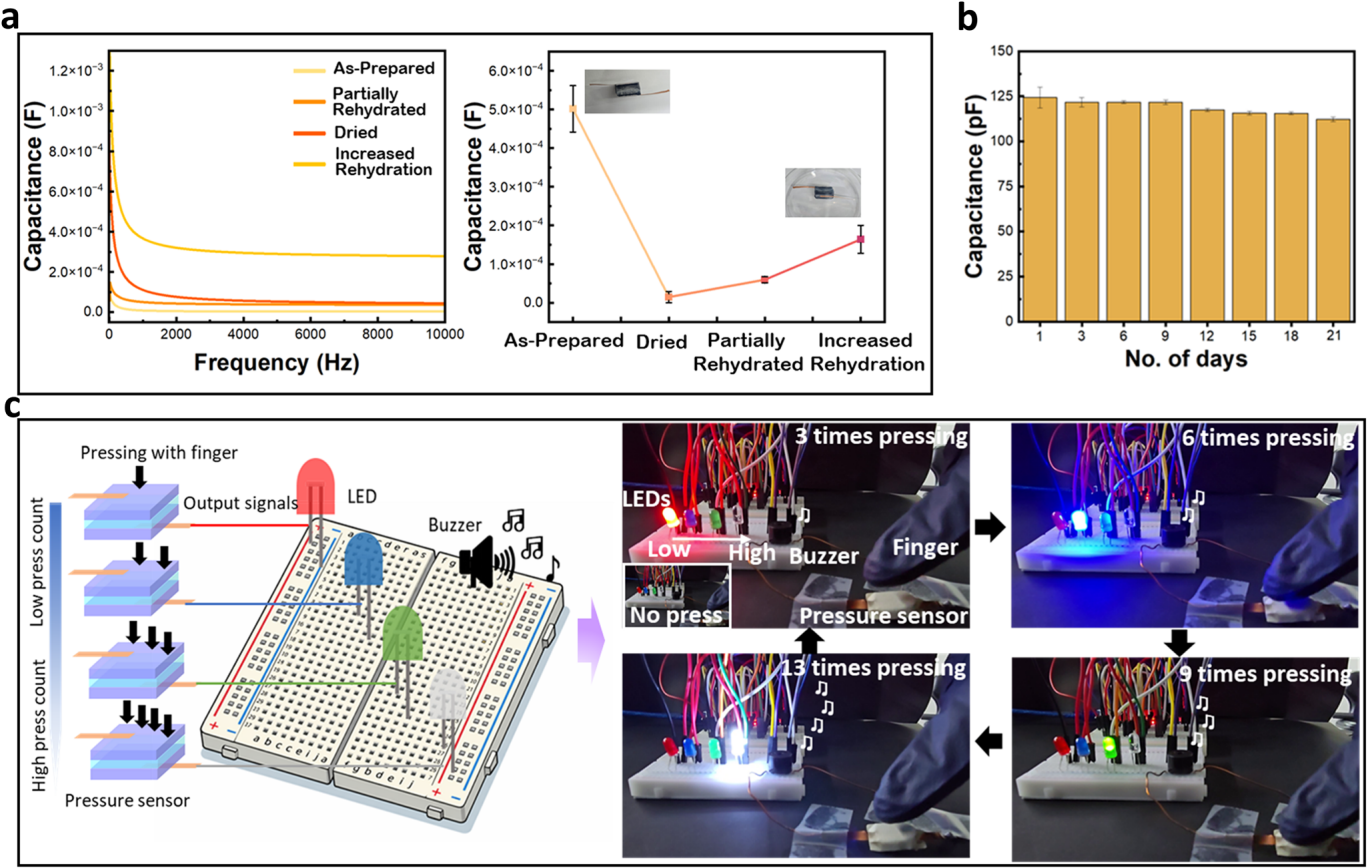
(a) Hydration‐dependent capacitance behavior of the hydrogel sensor. Capacitance variation with drying and rehydration; insets show representative photographs of the tested sensors. (b) Storage stability of the pressure sensor over 21 days. (c) Pressure sensor integrated with LED and sound buzzer pressed for HMI applications under different touch inputs.

The internal moisture may affect ionic transport within the hydrophilic PEGDA network. The impact of the matrix hydration state was tested under identical frequency parameters. The properties were monitored across four states: as‐prepared, fully dried, partially rehydrated, and increased rehydration. In the as‐prepared state, the swollen polymer mesh contains a large volume of free water molecules, facilitating ion migration and yielding a maximum capacitance of ∼0.5 mF (Figure 7a). Upon being completely dried, the water molecules force the polymer networks to collapse. This structural collapse drops the baseline capacitance near zero (0.02 mF). Remarkably, when the dehydrated matrix is exposed to moisture (Partially Rehydrated and Increased Rehydration states), the hydrophilic network absorbs water. This water uptake re‐expands the internal network, recovering the capacitance back toward its near original magnitude. This reversible response indicates that while the absolute capacitance values are dynamically sensitive to environmental moisture, the chemical crosslinks remain stable, providing a durable platform for wearable application.

The long‐term stability of the capacitive pressure sensor was assessed by monitoring its capacitance response over a period of 21 days, as presented in Figure 7b. The sensor exhibits a nearly constant capacitance value with only a slight decrease from 125 pF to 112 pF, indicating minimal signal drift over time. The minor reduction in capacitance may be due to gradual dehydration of the hydrogel matrix. Importantly, the overall stable response confirms the robustness of the sensor and its suitability for long‐term applications in wearable and flexible electronics. The environmental stability of the capacitive pressure sensor was evaluated by monitoring its frequency‐dependent dielectric response over three consecutive days under ambient conditions. The unencapsulated device (Figure S12a) exhibited a noticeable decline in dielectric constant by Day 3, particularly at low frequencies, owing to progressive moisture loss. In contrast, the encapsulated sensor (Figure S12b) showed minimal dielectric variation over the same period. The preservation of the low‐frequency response confirms that encapsulation effectively decreases dehydration and stabilizes the internal ionic environment, for the long‐term operational reliability of the sensor.

### 2.7 Real‐Time Human‐Machine Interfacing

To demonstrate the practical applicability, a real‐time HMI system was constructed by integrating the device with LED indicators and a sound buzzer (Figure 7c). Figure S13 shows the Arduino controlled wiring and circuit diagram for LED and buzzer setup. By design, the system was programmed to execute distinct feedback responses based on the number of loading cycles detected by the sensor. Upon mechanical triggering via manual pressing, the sensor generates a capacitance signal that is translated by the microcontroller into designated commands. As a proof‐of‐concept purpose, when three initial sequential presses were registered, the system activates a red LED and a baseline buzzer tone. As the number of input repetitions increases, the system shifts to illuminate a blue LED while simultaneously increasing the audio frequency of the buzzer feedback. When the input count increases to 6 sequential presses, the system shifts to illuminate a blue LED while simultaneously increasing the audio frequency of the buzzer. Upon reaching a higher number of inputs (9 and 13), the interface activates green and blue LEDs (Video S2). This multi‐triggered responsive behavior highlights the sensor's reliable signal generation and converts them into real‐time feedback signals, which allow a microcontroller to categorize mechanical stimuli. Such integration demonstrates the potential of the hydrogel‐based capacitive sensor for wearable electronics, smart interfaces, robotics and tactile sensing systems.

## 3 Experimental Section

### 3.1 Materials

All of the reagents were used in the experiments with analytical pure grade. PEGDA (700 Mn) as supplied by Sigma. Iron(III) p‐toluenesulfonate (FeTOS) was obtained from Sigma‐Aldrich. Imidazole was obtained from TCI. Phosphate‐buffered saline (PBS; 10X) consists of 137 mm NaCl, 2.7 mm KCl, and 10 mm PBS with pH 7.2. FITC‐dextran was purchased from Sigma‐Aldrich for fluorescence labelling. PBS buffer at pH 7.2 was used for chemical dissolution and as an electrolyte for all the measurement tests. All solutions were prepared using DI ultrapure water. All electrochemical experiments were performed with an Autolab potentiostat (PGSTAT128N, ECO CHEMIE BV, The Netherlands). A three‐electrode setup consisting of steel mesh (geometric area of 0.5 cm^2^), platinum wire, and Ag/AgCl– as the working, counter, and reference electrodes, respectively, was used for all experiments.

### 3.2 Preparation of Janus PEDOT/PEGDA Hydrogel

The Janus hydrogel was fabricated using a layer‐by‐layer casting approach. For the conductive hydrogel, an 18% (w/v) PEGDA solution was prepared in 1 mm PBS and mixed with 20% stock ammonium persulfate (APS). In parallel, EDOT was dissolved in 5% (v/v) ethanol, and oxidative polymerization was initiated by adding 1.5 m FeTOS) and 0.1 m H_2_SO_4_, allowing the reaction to proceed overnight at room temperature. The resulting PEDOT dispersion was added to the PEGDA solution and sonicated for 5 min to ensure homogeneity, followed by the addition of 0.15% TEMED to initiate gelation. For Janus assembly, 2 mL of the conductive precursor was cast into a mold and allowed to partially solidify for 5 min. A dielectric layer consisting of PEGDA prepared in deionized water was then added and cured for 5 min, followed by casting of the top conductive layer. The fully formed Janus hydrogel was removed from the mold and cut into rectangular samples (1.5 cm × 1.0 cm) for subsequent experiments.

### 3.3 Fabrication of Pressure Sensor

For complete fabrication of the pressure sensor, copper tape electrodes were attached to both outer conductive surfaces of the hydrogel system to serve as electrical contacts. The device was mechanically stabilized and electrically insulated by wrapping with adhesive tape, ensuring consistent contact during compression. Later, the whole device was encapsulated into PE plastic pouch using food packaging vacuum machine.

### 3.4 Characterizations

The surface of hydrogel was characterized by Cryo Scanning Electron Microscope‐CroSEM (FEI Quanta 200/Quorum PP2000TR FEI). Before SEM examination, all gels were dried using liquid nitrogen. To remove water from the samples an overnight freeze‐drying was performed. Then hydrogel samples were kept in liquid nitrogen and used directly for SEM. The fluorescence microscope (Olympus IX81 fluorescence microscope, Japan) was used to record fluorescence images. The electrical conductivity of conducting hydrogels were measured using 4‐point probe measuring instrument (Keithley 2400 SourceMeter with home‐made stage). The electrochemical measurements were recorded using a standard three‐electrode setup. Capacitance and impedance signals under applied pressure were recorded using an LCR meter (Keysight, E4980A) for all electrical measurements. The tensile properties of the hydrogel were evaluated using a tensile‐compression testing system (JSV‐H1000, JISC Ltd., Japan) with a digital force gauge (HF‐10, ALGOL, Japan).

To ensure statistical reliability and reproducibility across all evaluated parameters, all data points for the swelling ratio and mechanical characterization represent the mean values obtained from a sample of *n* = 3 per experiments with error bars indicating the standard deviation of the mean.

### 3.5 On‐Body Physiological Monitoring

Wearable monitoring evaluations were conducted as proof‐of‐concept using a single healthy volunteer participant. To ensure device reproducibility, all on‐body experiments were validated using a batch of three independently prepared Janus hydrogel sensor devices (*n* = 3). For monitoring, the integrated sensors were affixed to the skin surface (such as index finger joint or wrist) utilizing a thin, biocompatible medical‐grade adhesive tape. All real‐time electromechanical tracking trials were carried out under controlled, ambient laboratory conditions (24°C–26°C) and 50%–55% RH to isolate the sensor response from atmospheric fluctuations.

### 3.6 Ethical Statement

The human evaluation protocols in this study were reviewed and approved by the Institutional Review Board for Biomedical Science Research, Academia Sinica (AS‐IRB‐BM). The study was conducted in accordance with the ethical principles. Human participant volunteered voluntarily and provided written informed consent prior to the physiological monitoring experiments.

## 4 Conclusion

In this work, a monolithic Janus hydrogel‐based capacitive pressure sensor was successfully designed for wearable motion and physiological monitoring. By integrating conductive polymer (PEDOT) and dielectric functionalities within a single continuous hydrogel (PEGDA) architecture, the proposed design effectively overcomes the limitations of conventional multilayer structures, including interfacial delamination, mechanical instability, and fabrication complexity. The sensor was fabricated using a simple and scalable approach, enabling precise control over material composition and structural properties. Systematic investigations demonstrated that key parameters such as PEDOT concentration, glycerol content, PEGDA composition, and dielectric thickness play critical roles in tuning the electrical and mechanical performance of the device.

As a result, the obtained sensor exhibited a fast response rate of 23 ms and recovery rate of 36 ms, together with a wide working pressure range (0.49– 5.39 kPa) and cycling stability upto 3000 cycles. Furthermore, the sensor has the potential to be used for human motion and healthcare monitoring, including recognition of facial expressions, joint bending, and pressure distribution. Overall, the monolithic pressure sensor presents a robust, sensitive, and versatile platform for wearable electronics. Future work may focus on integrating wireless modules and multi‐sensor arrays to further expand its functionality in real‐world applications.

## Author Contributions

**Syed Atif Ali**: conceptualization, methodology, writing – review and editing, investigation, data curation, formal analysis, validation, visualization, writing – original draft. **Hsiung‐Lin Tu**: funding acquisition, supervision, writing – review and editing, validation, conceptualization, visualization, project administration, resources, writing – original draft. **Yu‐Sheng Hsiao**: methodology, formal analysis, writing – review and editing. **Reynaldo Montalbo**: formal analysis, writing – review and editing, validation. **Chih‐Wei Chu**: funding acquisition, resources, writing – review and editing. **Hailemichael Ayalew**: validation, writing – review and editing, methodology. **Zeeshan Alam Ansari**: conceptualization, methodology, writing – review and editing, investigation, validation, data curation, visualization. **Hsiao‐hua Yu**: resources, funding acquisition, writing – review and editing.

## Conflicts of Interest

The authors declare no conflicts of interest.

## Supporting information




**Supporting File 1**: advs76893‐sup‐0001‐SuppMat.docx.

**Supporting File 2**: advs76893‐sup‐0002‐FigureS1‐S13.zip.

**Supporting File 3**: advs76893‐sup‐0003‐VideoS1‐S2.zip.

## Data Availability

The data that support the findings of this study are available on request from the corresponding author. The data are not publicly available due to privacy or ethical restrictions.
