# Supplementary material for: A Monolithic Janus Hydrogel Pressure Sensor for Wearable Motion and Physiological Monitoring
Source: Adv Sci (Weinh). 2026 Jul 30:e76893. Online ahead of print. doi: 10.1002/advs.76893 (PMC13423481; doi:10.1002/advs.76893)
Supplement: Supplementary file 1 — Supporting File 1: advs76893‐sup‐0001‐SuppMat.docx. [file ADVS-9999-e76893-s001.docx]

**Supporting Information**

**A Monolithic Janus Hydrogel Pressure Sensor for Wearable Motion and Physiological Monitoring**

Syed Atif Ali,^||a^ Zeeshan Alam Ansari,^||b,c,d^ Reynaldo Montalbo,^a^ Hailemichael Ayalew,^a,e^ Yu-Sheng Hsiao,^f^ Chih-Wei Chu,^c,d^ Hsiao-hua Yu,^a,e^ Hsiung-Lin Tu*^a^

*^a^Institute of Chemistry, Academia Sinica, Nankang, Taipei 11529, Taiwan*

*^b^Department of Physics, National Taiwan University, Sec. 4, Roosevelt Road, Taipei 106, Taiwan, Taiwan*

*^c^Nano-Science and Technology Program, Taiwan International Graduate Program, Academia Sinica, Taipei 106, Taiwan*

*^d^Research Center for Applied Sciences, Academia Sinica, 128, Academia Road, Section 2, Nangang, Taipei 11529, Taiwan*

*^e^Smart Organic Materials Laboratory, Institute of Chemistry, Academia Sinica, No. 128, Section 2, Academia Road, Nankang, Taipei, Taiwan*

*^f^Department of Materials Science and Engineering, National Taiwan University of Science and Technology, Taipei 106335, Taiwan*

*^*^Corresponding authors: hltu@gate.sinica.edu.tw*

**Experimental section**

**S1.1 Compressive strength of the samples**

The strain vs stress of the samples was estimated using a mechanical testing Rheometer. Cylindrical samples with 7 mm height (*h_0_*) and 10 mm diameter (*d*) dimensions were placed on a flat surface. The upper plate was programmed to compress the sample at a controlled, constant displacement rate. The normal force (*F*) exerted by the hydrogel network and the corresponding axial displacement (*Δh*) were continuously recorded by the instrument software. All measurements were conducted at ambient room temperature to ensure environmental consistency across tests.

The compressive stress is calculated by eq 1

1

$$\sigma=\frac{F}{A_{0}}=\frac{F}{\pi\cdot r^{2}}$$

F is the recorded force (N), *A_0_* is the initial cross-sectional area of the sample (m^2^) and r is the initial radius of the cylinder. compressive engineering strain is calculated as the ratio of the change in height to the initial pristine height of the cylinder with the eq 2.

2

$$\varepsilon(\%)=\frac{\Delta h}{h_{0}}\times100$$

*Δh* is the displacement change (mm), *h_0_* is the initial sample height.

**S1.2 Integration of the Hydrogel Sensor with the Microcontroller Interface**

To evaluate the real-time response, dynamic deformation speed, and multi-level pressure threshold discrimination capabilities of the designed monolithic Janus hydrogel sensor, an integrated hardware interface was constructed using an Arduino microcontroller platform **(Figure S9)**. The hydrogel capacitive sensor (Cs) was configured with resistor (R1) to form a low-pass resistor-capacitor network. The input terminal of the sensor circuit was driven by a digital I/O pin (Pin 4), while the intermediate node between the resistor and the hydrogel sensor was connected to an analog input channel (Pin A0) to monitor the voltage. The time constant derived from the charging duration was processed by the Arduino controller via a custom C++ program to calculate the instantaneous capacitance change (ΔC/C_0_) resulting from pressing the sensor. Along with amplitude mapping, the program was modified to retrieve changes and frequencies from the capacitance accumulated from sensor.

To demonstrate smart tactile response, the microcontroller was programmed to actuate a sequential indicator array consisting of a piezoelectric buzzer and four color-coded light-emitting diodes (LEDs). A 220 resistor (R2 to R6) also connected. The system distinguishes both loading rates and cyclic inputs: (i) a slow deformation rate triggers the activation of the red LED, (ii) whereas sequential tapping triggers further responses. Specifically, reaching a threshold of 6 compression cycles activates the blue LED, while a higher accumulation of 9 compression illuminates the green LED. The digitized sensor signals and threshold-activated events were transmitted via a USB interface to a host computer for real-time visualization, processing, through the Arduino controller interface.


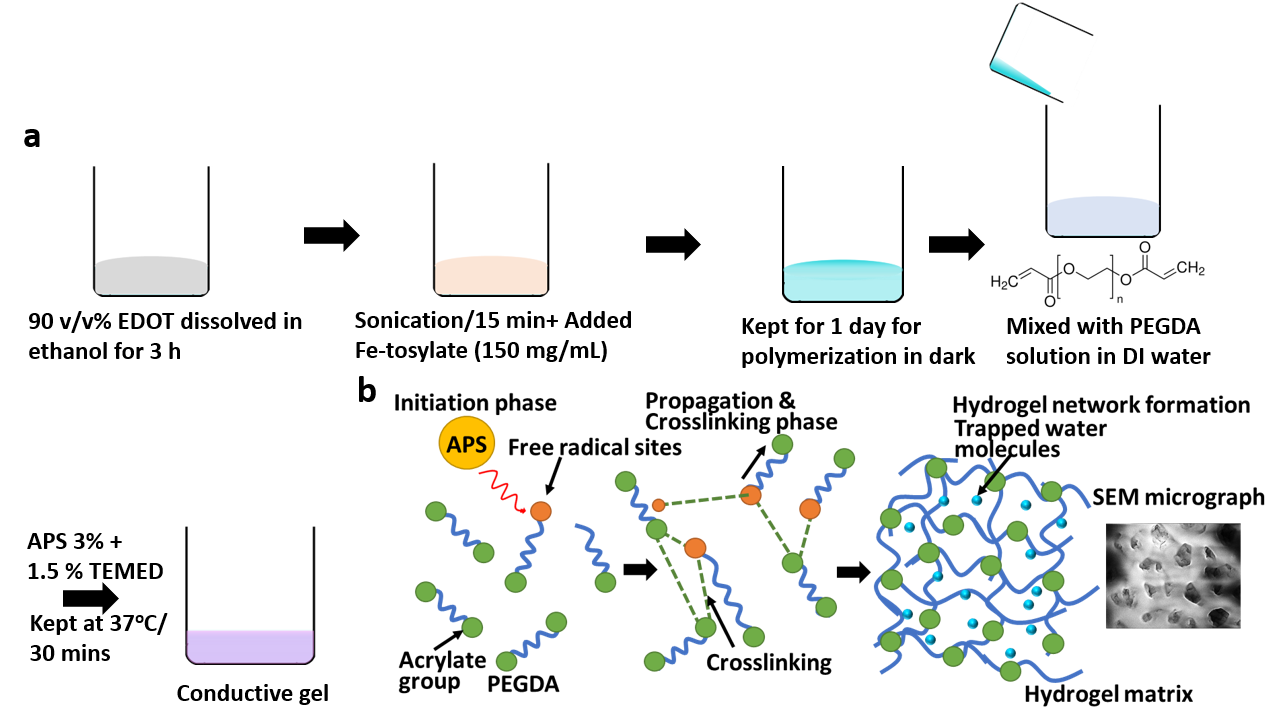


**Figure S1.** (a) Process of preparation of conductive hydrogel using oxidative polymerization method. (b) Mechanism of formation of hydrogel.


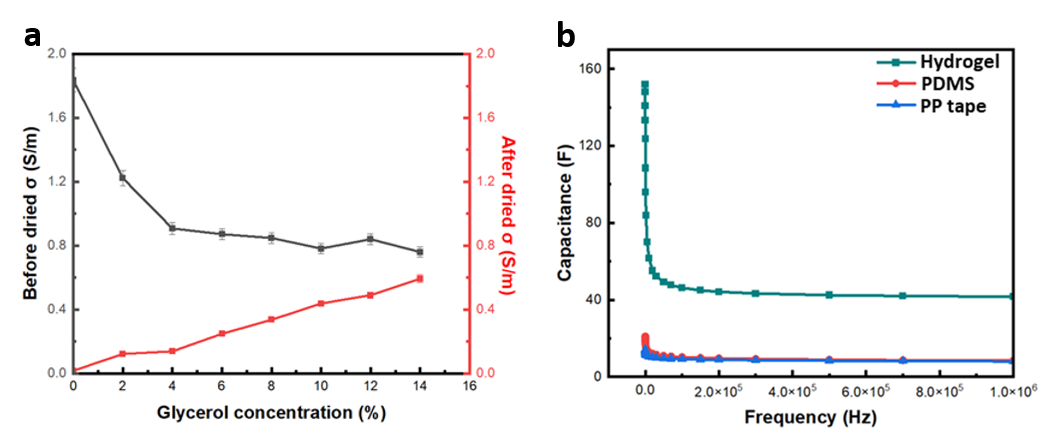


**Figure S2.** (a) Effect of glycerol concentration on the electrical conductivity (σ) of the conductive hydrogel before and after drying. (b) Comparison of capacitance as a function of frequency for PEGDA hydrogel, PDMS, and polypropylene (PP) tape.


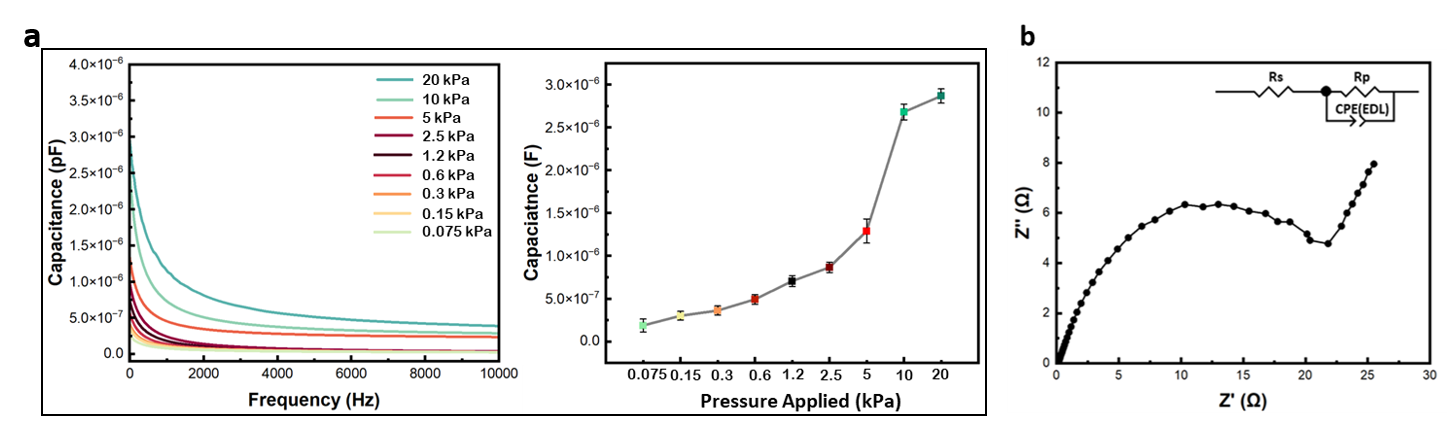


**Figure S3.** (a) Electromechanical mechanism validation of the Janus hydrogel sensor. Frequency curves across varied static pressures (0.075-20 kPa). (b) Nyquist plot with the corresponding equivalent circuit model used for quantitative impedance fitting.


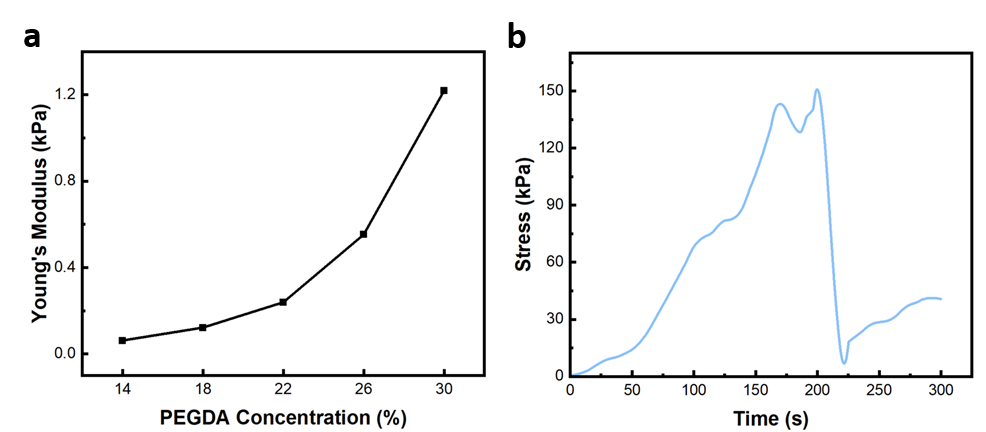


**Figure S4.** (a) Compressive Young's modulus of the PEGDA hydrogels as a function of polymer concentration (Linear fit range : 0-15%). (b) Time-dependent compressive stress relaxation profile of the PEGDA hydrogel under a sustained fixed strain.


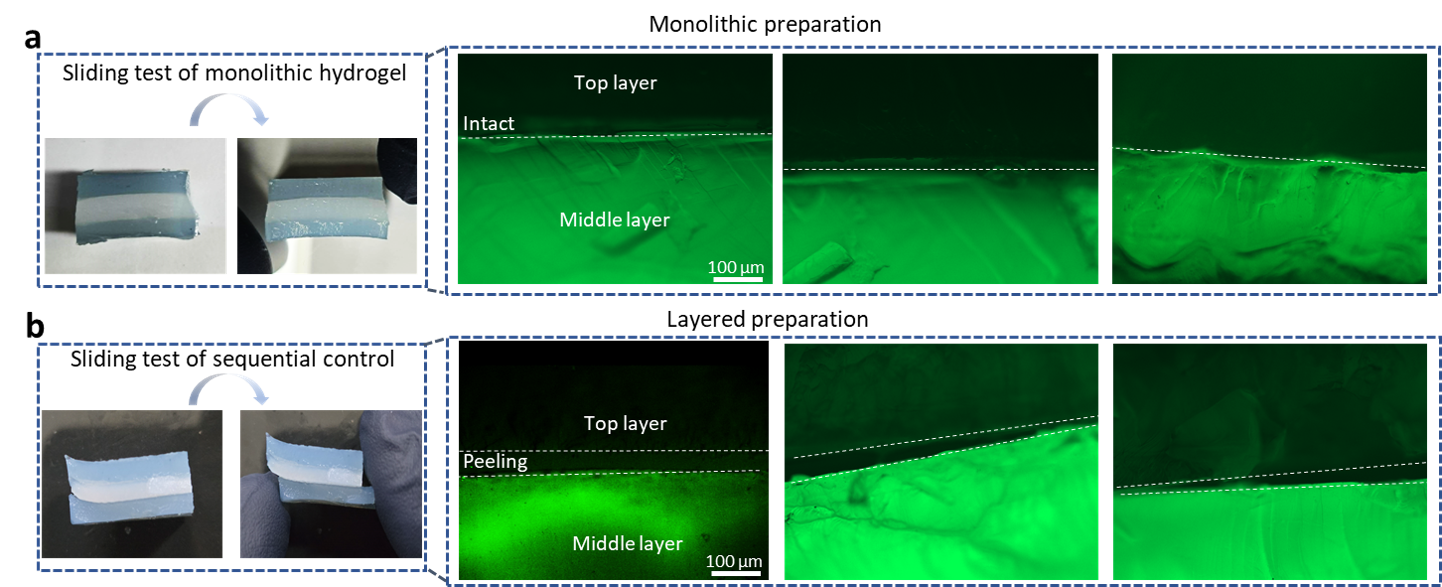


**Figure S5.** Comparative interfacial stability of Janus hydrogels under mechanical deformation. (a) Digital photographs and corresponding cross-sectional fluorescence images of the monolithic hydrogel architecture during a mechanical sliding test, showing an intact and continuous interface. (b) Digital photographs and fluorescence cross-sections of the sequentially layered control hydrogel (fabricated with a 10-minute polymerization delay) under identical testing conditions, displaying peeling at the layer boundary. Scale bars: 100 μm.


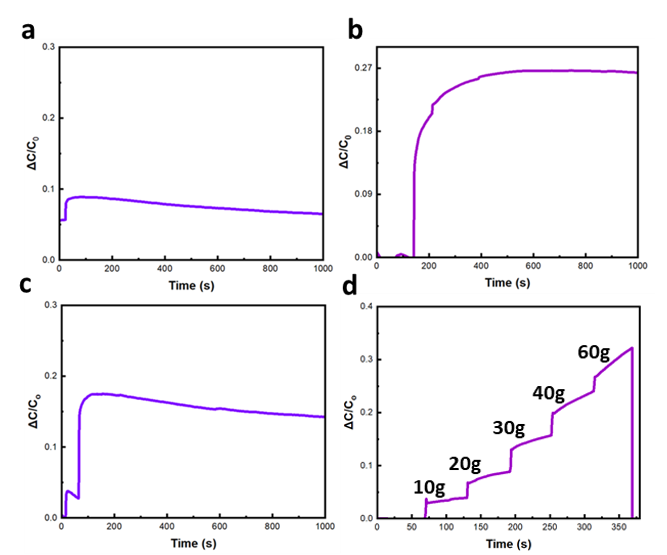


**Figure S6.** Experimental setup and pressure response characterization of the pressure sensor. (a-c) Normalized capacitance change (ΔC/C₀) recorded under a constant applied pressure over time, showing a rapid initial response followed by a stable plateau with minimal drift. (d) Stepwise pressure loading test with incremental pressure levels (0.2-1.4 kPa).


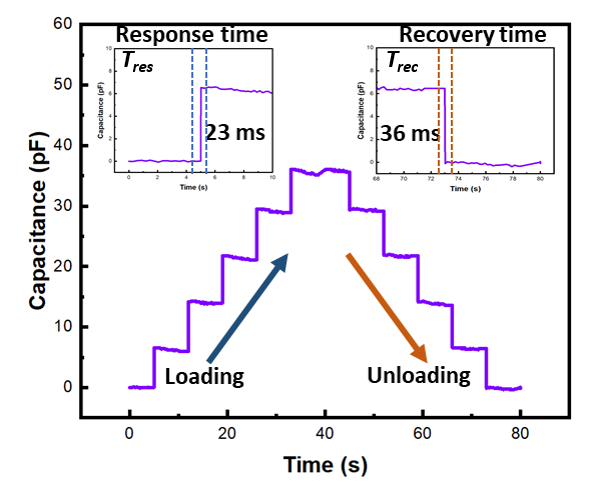


**Figure S7.** Graph depicting loading and unloading of known weight and in inset response and recovery time of the sensor.


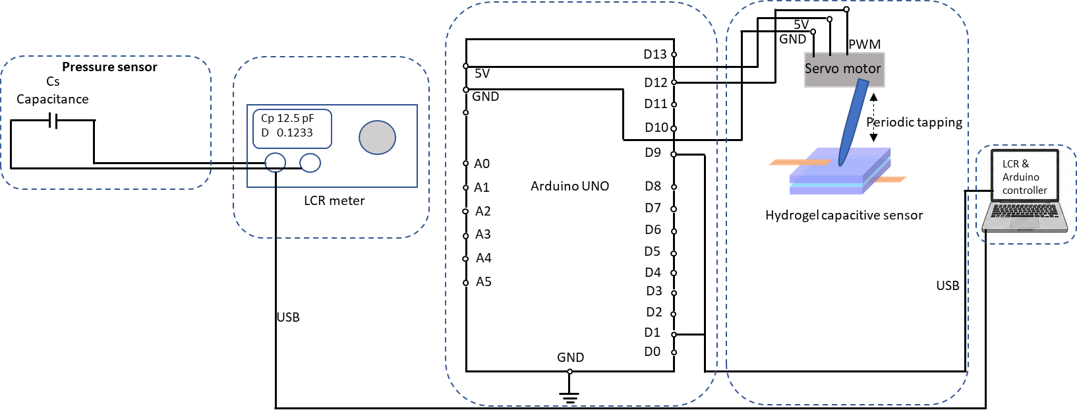


**Figure S8.** Schematic and circuit diagram to connect pressure sensor to Servo motor to Arduino.


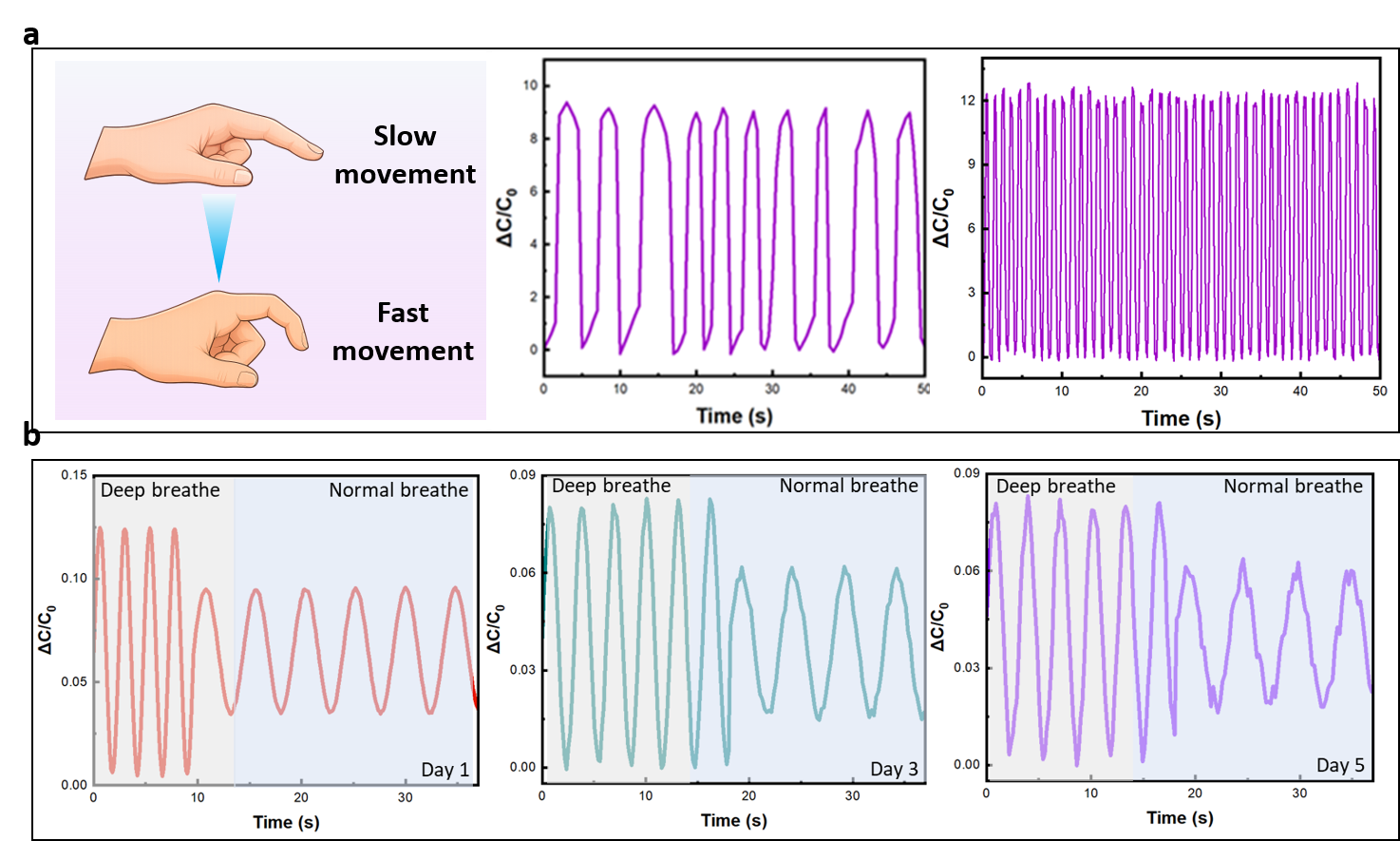


**Figure S9.** Wearable sensing performance of the sensor. (a) Finger bending detection at different bending angles (30°, 60°, and 90°), showing a progressive increase in normalized capacitance change (ΔC/C₀) with increasing bending angle, demonstrating the sensor’s ability to resolve different degrees of joint deformation. (b) Real-time respiration pattern recorded across multiple days, and periodic capacitance fluctuations correspond to rhythmic arterial pulsation, indicating physiological pressure variations. (Authors have used open-source AI platform (Chatgpt) to generate finger image in Figure a)


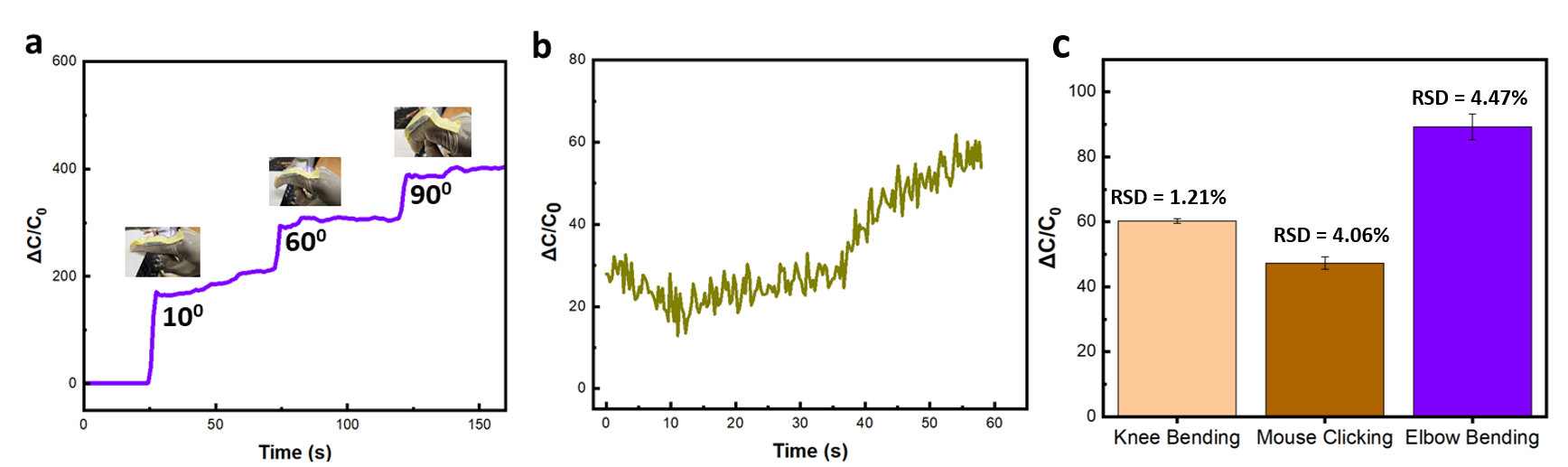


**Figure S10.** Wearable sensing performance of the sensor. (a) Finger bending detection at different bending angles (10°, 60°, and 90°), showing a progressive increase in normalized capacitance change (ΔC/C₀) with increasing bending angle. (b) Real-time pulse monitoring from the human hand, periodic capacitance fluctuations correspond to rhythmic arterial pulsation. (c) Statistical device-to-device reprodicibility evaluation (n = 3 independent sensor replicates).


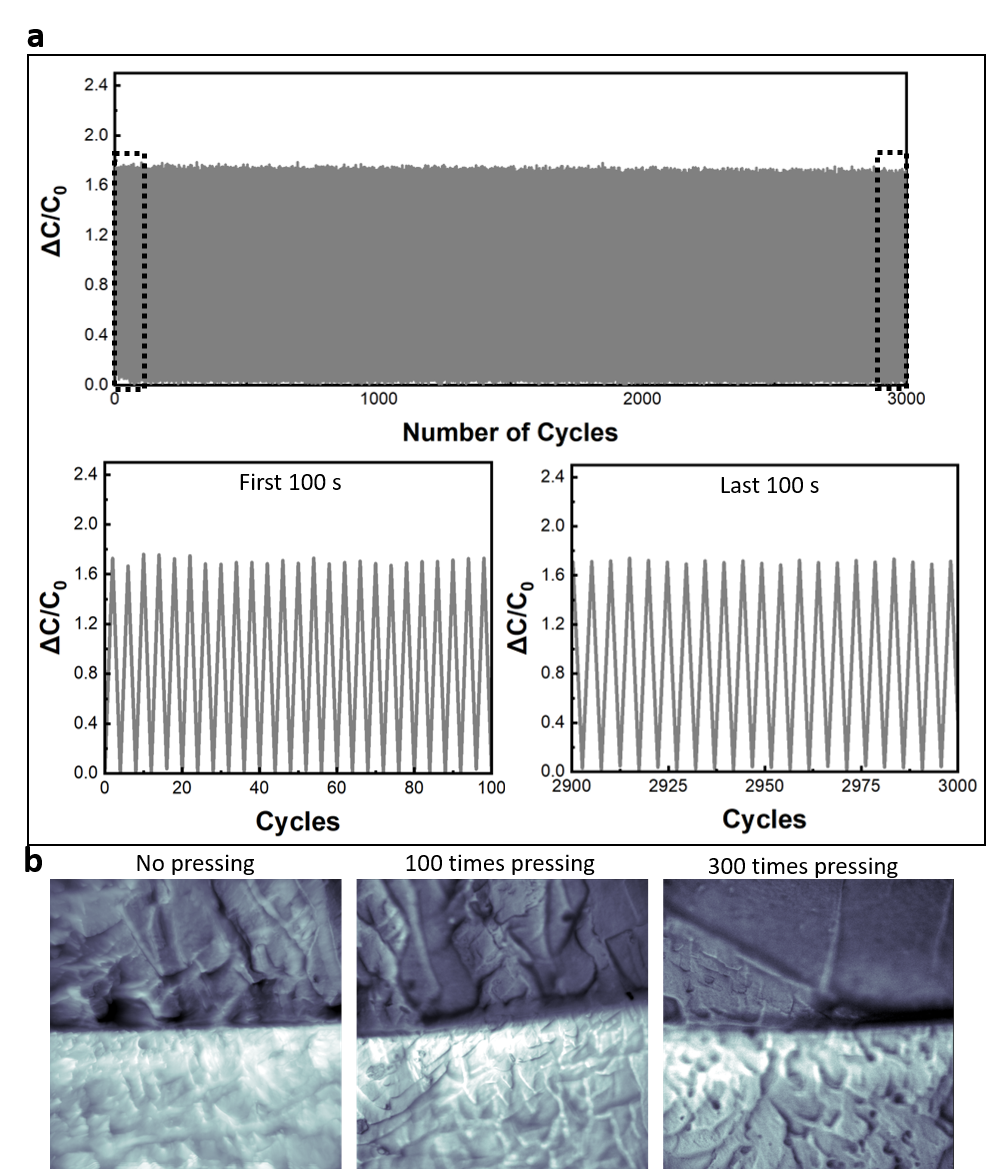


**Figure S11.** (a) Long-term stability of the Janus hydrogel pressure sensor under continuous cyclic loading. Normalized capacitance change (ΔC/C₀) recorded over ~3000 cycles during repeated pressing-releasing cycles, showing stable signal output with only minor amplitude attenuation. Enlarged view of the first and last 100 s, demonstrating consistent peak amplitude and waveform shape at the initial stage of operation. (b) Cross-sectional optical microscopy images of the Janus hydrogel sensor interface demonstrating the structural stability of the Janus hydrogel sensor interface. The continuous junction boundary in its pristine state, after 100 pressings, and after 300 pressings cycle.


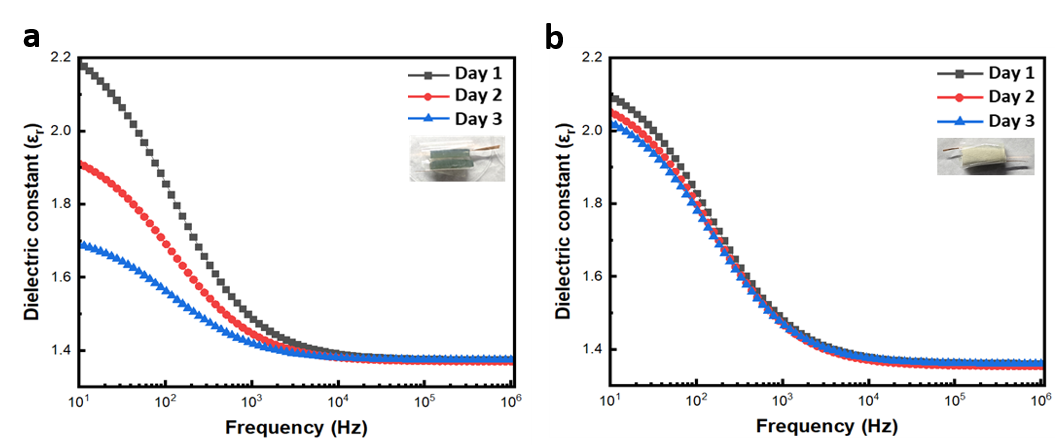


**Figure S12.** Stability of the capacitive pressure sensor under ambient conditions. Frequency-dependent capacitance measured on Day 1, Day 2, and Day 3, over time (a) without encapsulation and (b) with encapsulation of sensor.


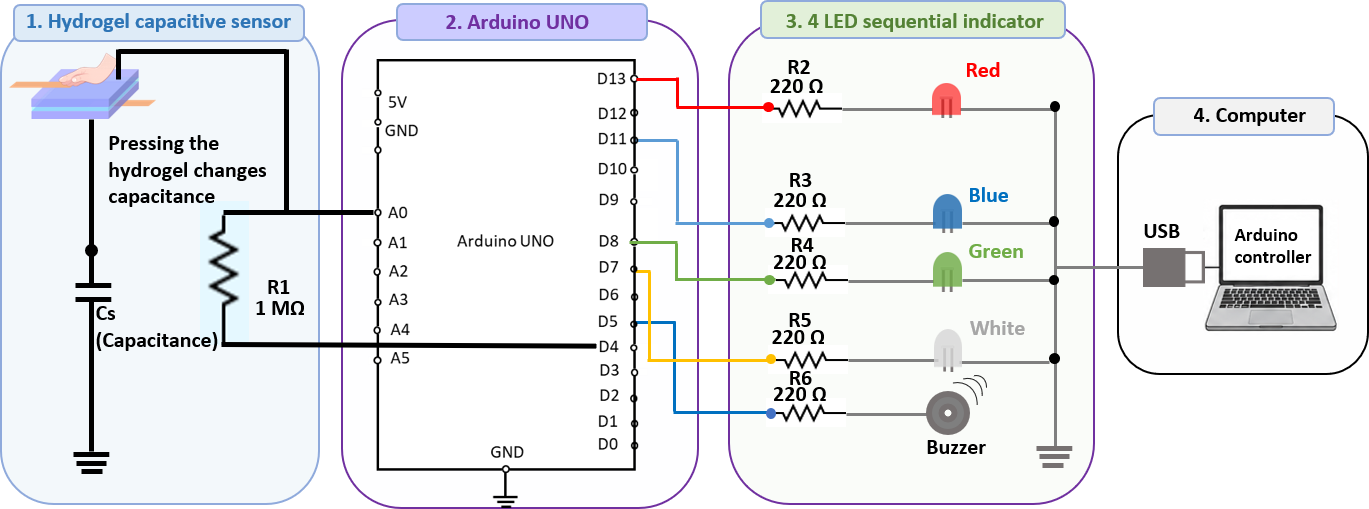


**Figure S13.** Schematic and circuit diagram to connect pressure sensor to LED and buzzer to Arduino.

| **Circuit Element** | **Values** |
| --- | --- |
| Rs | 60.8 Ω |
| Rp | 6181 Ω |
| CPE1-T | 1.514 x 10-5 F |
| CPE1-P | 0.675 |

**Table S1.** Fitted equivalent circuit parameters obtained from the electrochemical impedance spectroscopy (EIS) data of the Janus hydrogel sensor.

**Table S2.** Comparison of sensing performance parameters of the deve;loped pressure sensor with previously reported pressure sensors.

| **Pressure sensor type** | **Sensing Mechanism** | **Response time/ Recovery time (ms)** | **Sensitivity**  **(kPa^-1^)** | **Working Range (kPa)** | **Durability (Cycles)** | **Ref** |
| --- | --- | --- | --- | --- | --- | --- |
| CNT/MXene/PDMS electrodes | Capacitive | 71 | 0.091 | 1 Pa – 2 MPa | 6000 | S1 |
| Nickel/Polyimide (Ni/PI) composite nanofiber membrane | Capacitive | 30 | 0.00404 | 0-1.5 MPa | 1000 | S2 |
| nylon netting spacer in a sandwich-like structure | Capacitive | <20 | 0.33 | 0-1 kPa | >1000 | S3 |
| PVA/cupric sulfate hydrogel with graphene | Capacitive | - | 0.0491 (0–100 kPa),0.0180 (100–300 kPa),0.0100 (300–600 kPa),0.0041 (0.6–1 MPa) | 0-1 MPa | 1000 | S4 |
| PDMS skeleton with a Sodium Alginate (SA) hydrogel core | Capacitive | 45/70 | 14.25 | 400 | - | S5 |
| CNT-doped Ecoflex porous nanocomposite (PNC) | Hybrid | - | 3.13 (Low-P)  0.43 (High-P) | 0-1/30-50 | 5000 | S6 |
| PVA/Chitosan dual-network ionic hydrogel | Capacitive | 11 |  | 0-290 | >2000 | S7 |
| **Janus hydrogel** | Capacitive | **23/36** | **0.27 (Low-P),** **0.05 (High-P)** | **0.49-3.43/3.43-5.39** | **3000** | **This work** |

**References**

S1. X. Y. Li, Y. N. Liu, Y. R. Ding, M. Zhang, Z. H. Lin, Y. Hao, Y. C. Li, J. J. Chang, Capacitive Pressure Sensor Combining Dual Dielectric Layers with Integrated Composite Electrode for Wearable Healthcare Monitoring *Acs Appl Mater Inter*, *16* (2024): 12974- 12985.

S2. L. T. Xia, W. Xiao, L. X. Li, X. Liu, Q. B. Zhuang, Y. Huang, T. H. Lan, X. H. Du, Y. Zhao, D. Z. Wu, “High-Performance Flexible Capacitive Pressure Sensor Based on a Spiked Nickel/Polyimide Composite Nanofiber Membrane,” *ACS Sensors* 10 (2025): 1450-1460.

S3. Z. F. He, W. J. Chen, B. H. Liang, C. Y. Liu, L. L. Yang, D. W. Lu, Z. C. Mo, H. Zhu, Z. K. Tang, X. C. Gui, “Capacitive Pressure Sensor with High Sensitivity and Fast Response to Dynamic Interaction Based on Graphene and Porous Nylon Networks,” *ACS Applied Materials & Interfaces* 10 (2018): 12816-12823.

S4. M. Chen, S. Ghorbanzadeh, W. Zhang, A low-energy-dissipating hydrogel-based capacitive sensor for therapeutic pressure monitoring Sensor Actuat a-Phys 2025, 395.

S5. H. Z. Huang, X. Ran, S. Wan, Y. Wang, H. C. Bi, “A flexible capacitive pressure sensor with a hybrid porous PDMS/SA hydrogel structure for touch/pain detection,” *Nanoscale* 16 (2024): 17926-17933.

S6. K. H. Ha, W. Y. Zhang, H. Jang, S. M. Kang, L. Wang, P. Tan, H. Hwang, N. S. Lu, Highly Sensitive Capacitive Pressure Sensors over a Wide Pressure Range Enabled by the Hybrid Responses of a Highly Porous Nanocomposite Adv Mater 2021, 33 (48),

S7. C. X. Liu, F. Ma, Q. C. Sun, Q. S. Hu, W. Tong, X. Guo, R. H. Hu, P. Liu, Y. Huang, X. T. Hao, W. Z. Ma, Y. G. Zhang, Highly Sensitive Flexible Capacitive Pressure Sensor Based on a Multicross-Linked Dual-Network Ionic Hydrogel for Blood Pressure Monitoring Applications Acs Appl Mater Inter 2024, 16 (26), 34042,
