# Supplementary figures and images for: A Monolithic Janus Hydrogel Pressure Sensor for Wearable Motion and Physiological Monitoring
Source: Adv Sci (Weinh). 2026 Jul 30:e76893. Online ahead of print. doi: 10.1002/advs.76893 (PMC13423481; doi:10.1002/advs.76893)

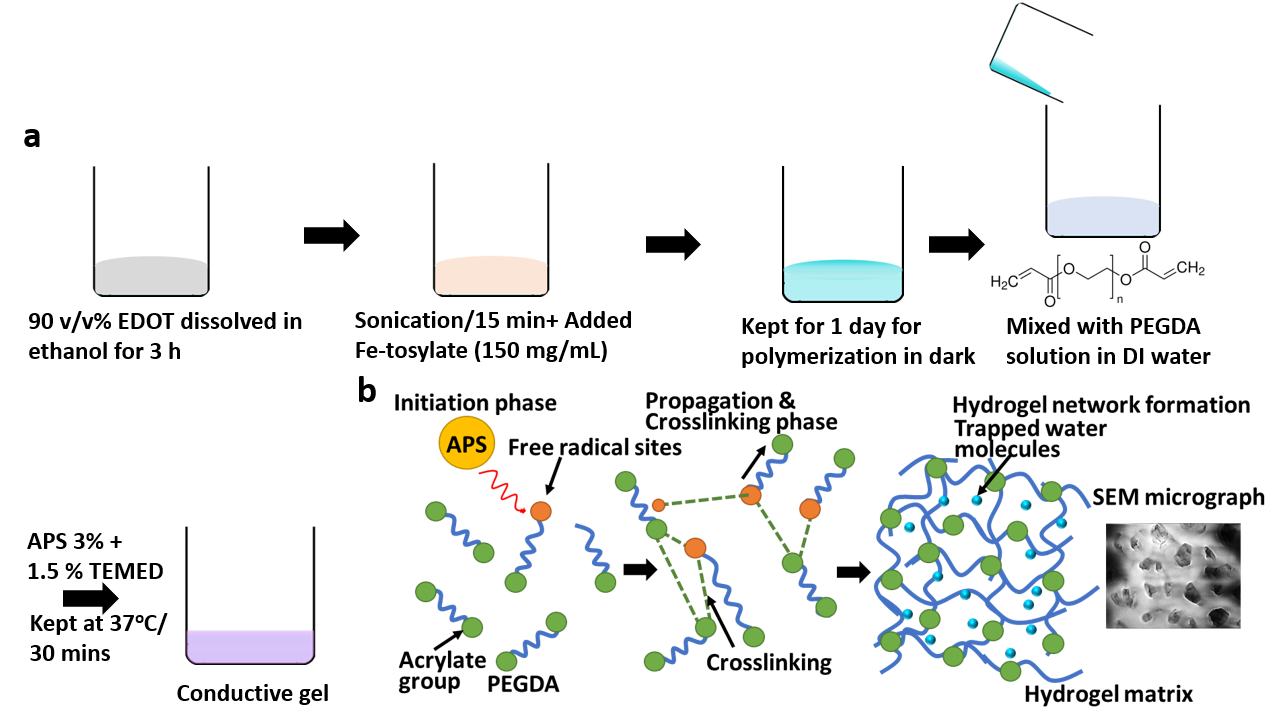

Supplement: Supplementary file 2 — Supporting File 2: advs76893‐sup‐0002‐FigureS1‐S13.zip. [file ADVS-9999-e76893-s002.zip › Figure S1.tif]

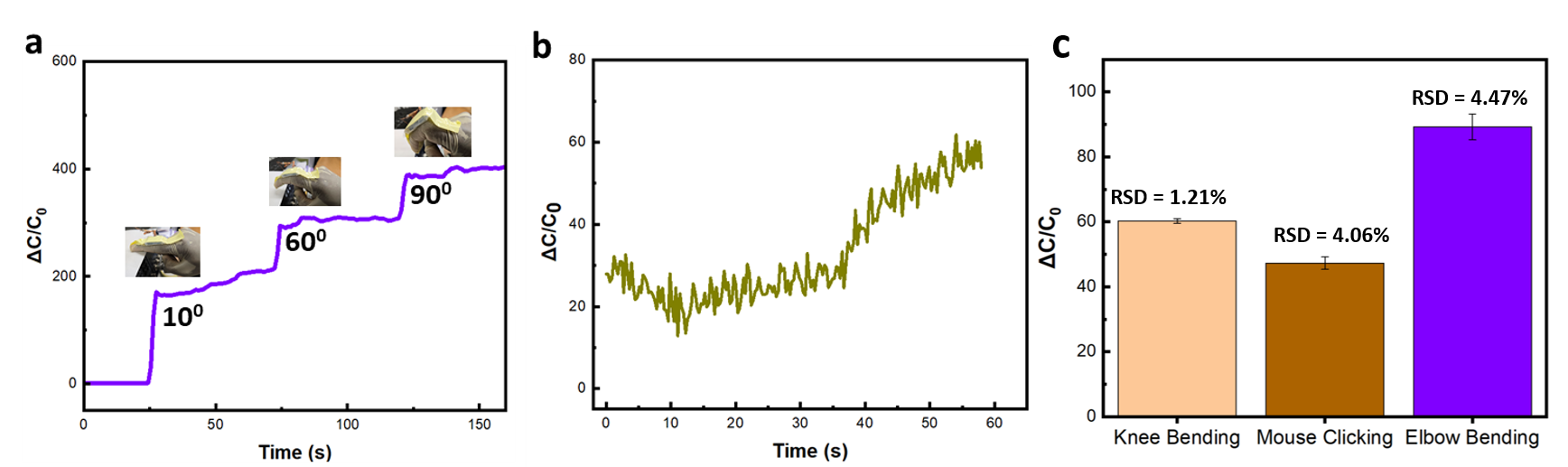

Supplement: Supplementary file 2 — Supporting File 2: advs76893‐sup‐0002‐FigureS1‐S13.zip. [file ADVS-9999-e76893-s002.zip › Figure S10.tif]

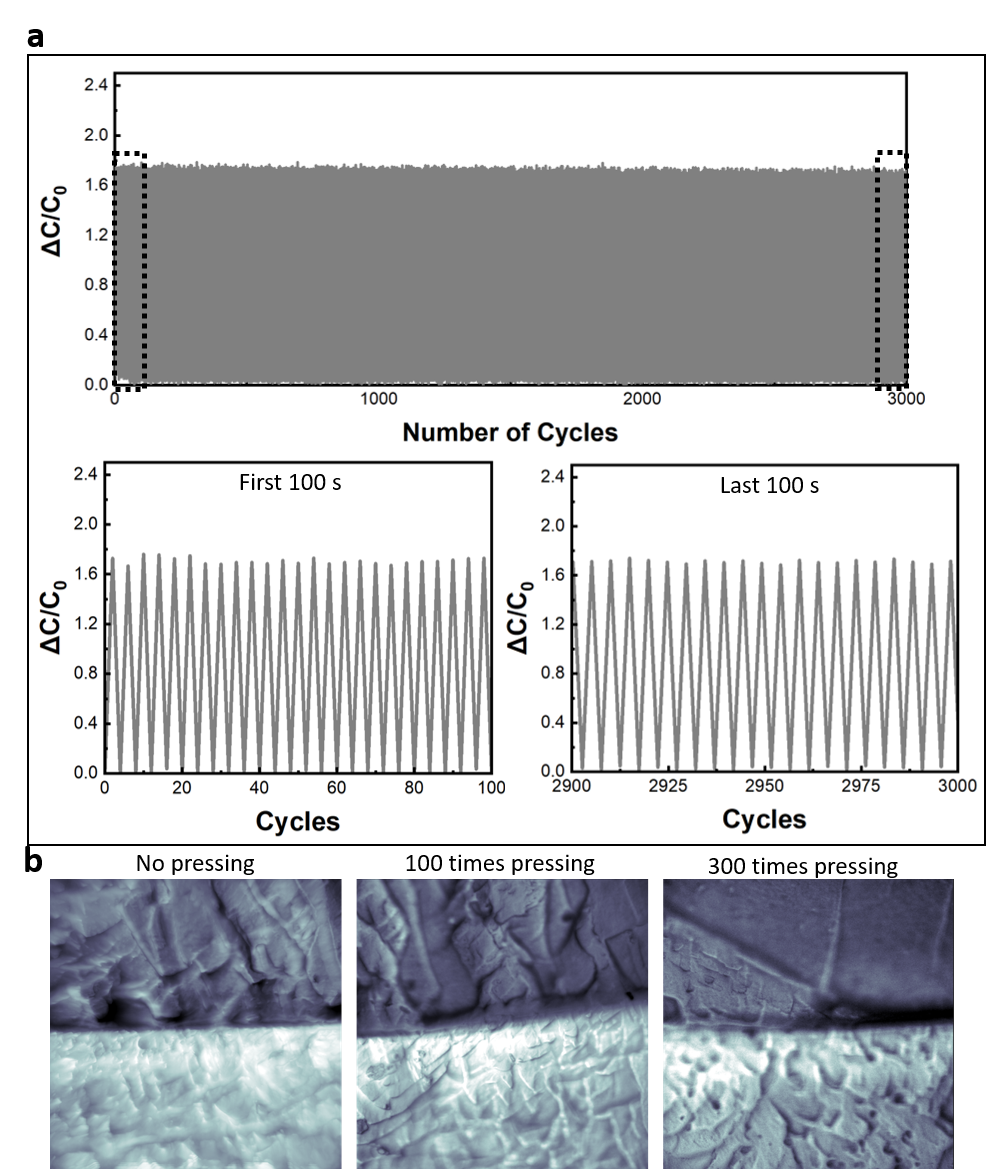

Supplement: Supplementary file 2 — Supporting File 2: advs76893‐sup‐0002‐FigureS1‐S13.zip. [file ADVS-9999-e76893-s002.zip › Figure S11.tif]

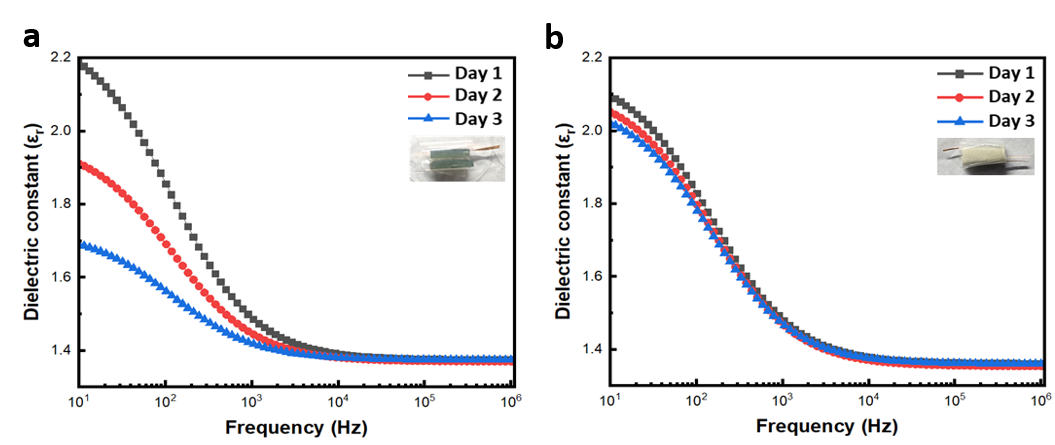

Supplement: Supplementary file 2 — Supporting File 2: advs76893‐sup‐0002‐FigureS1‐S13.zip. [file ADVS-9999-e76893-s002.zip › Figure S12.tif]

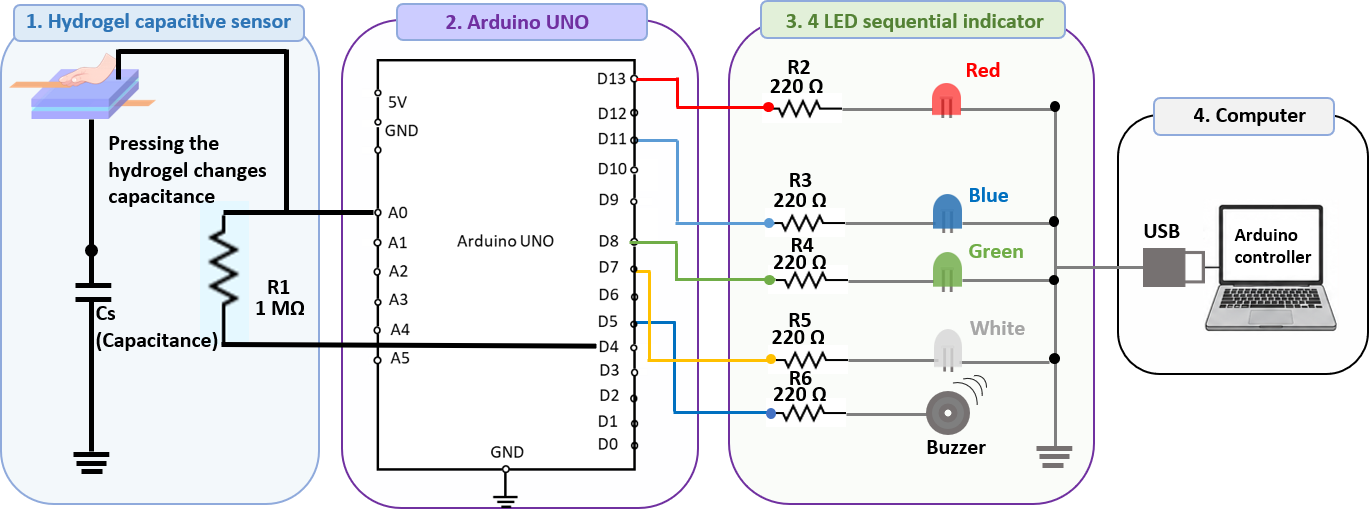

Supplement: Supplementary file 2 — Supporting File 2: advs76893‐sup‐0002‐FigureS1‐S13.zip. [file ADVS-9999-e76893-s002.zip › Figure S13.tif]

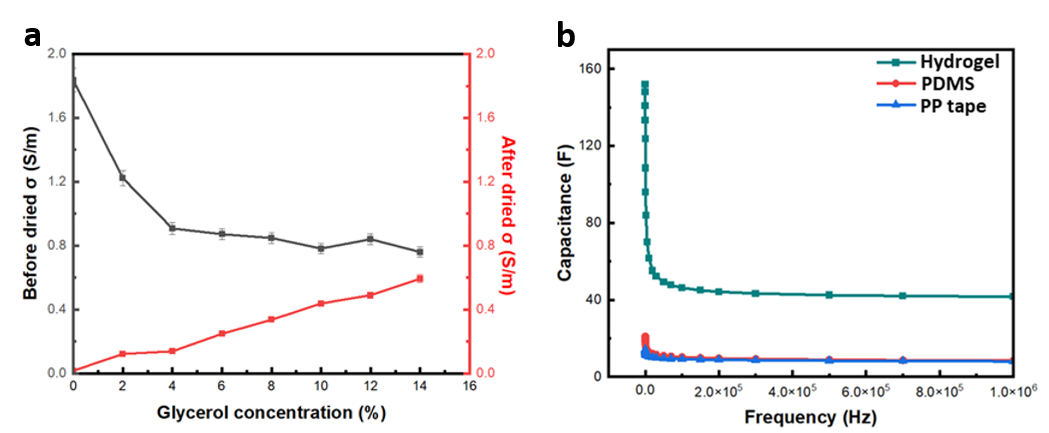

Supplement: Supplementary file 2 — Supporting File 2: advs76893‐sup‐0002‐FigureS1‐S13.zip. [file ADVS-9999-e76893-s002.zip › Figure S2.tif]

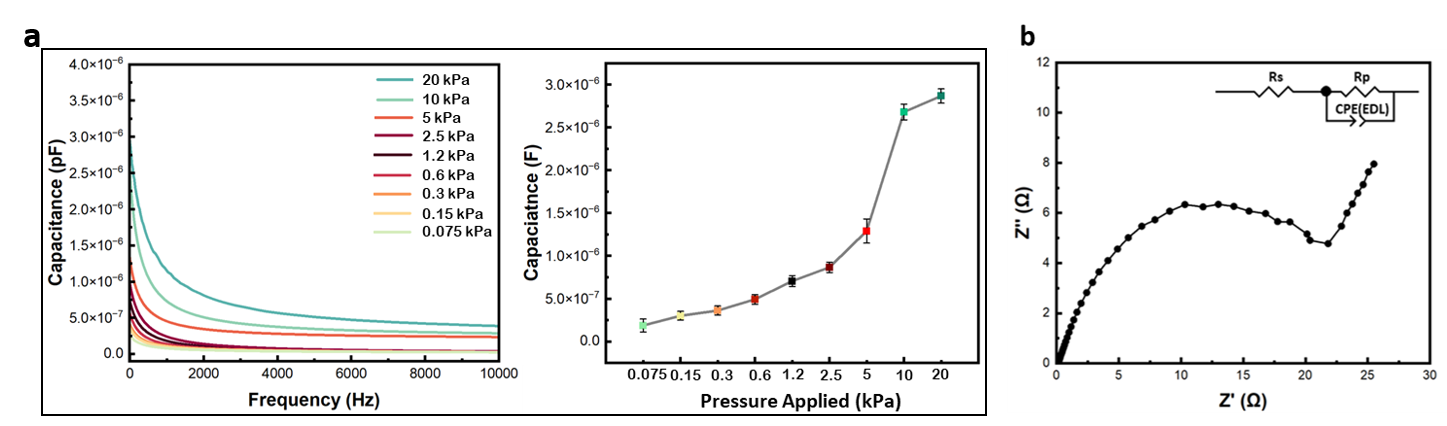

Supplement: Supplementary file 2 — Supporting File 2: advs76893‐sup‐0002‐FigureS1‐S13.zip. [file ADVS-9999-e76893-s002.zip › Figure S3.tif]

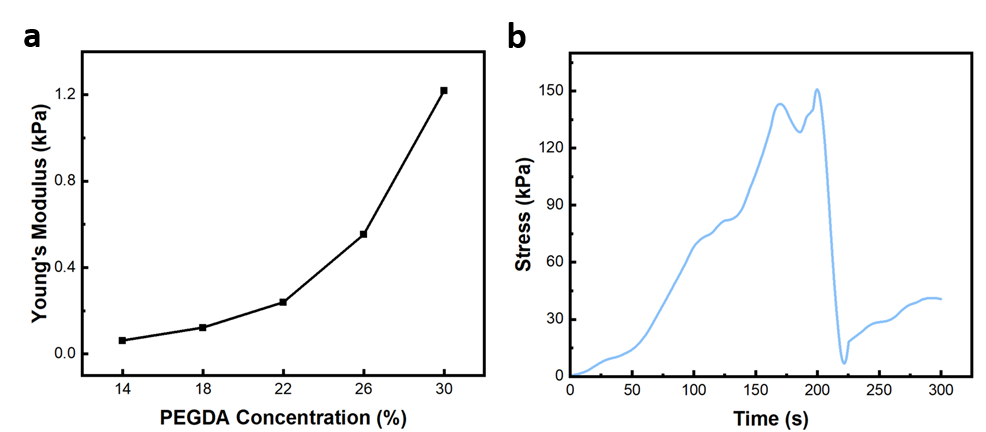

Supplement: Supplementary file 2 — Supporting File 2: advs76893‐sup‐0002‐FigureS1‐S13.zip. [file ADVS-9999-e76893-s002.zip › Figure S4.tif]

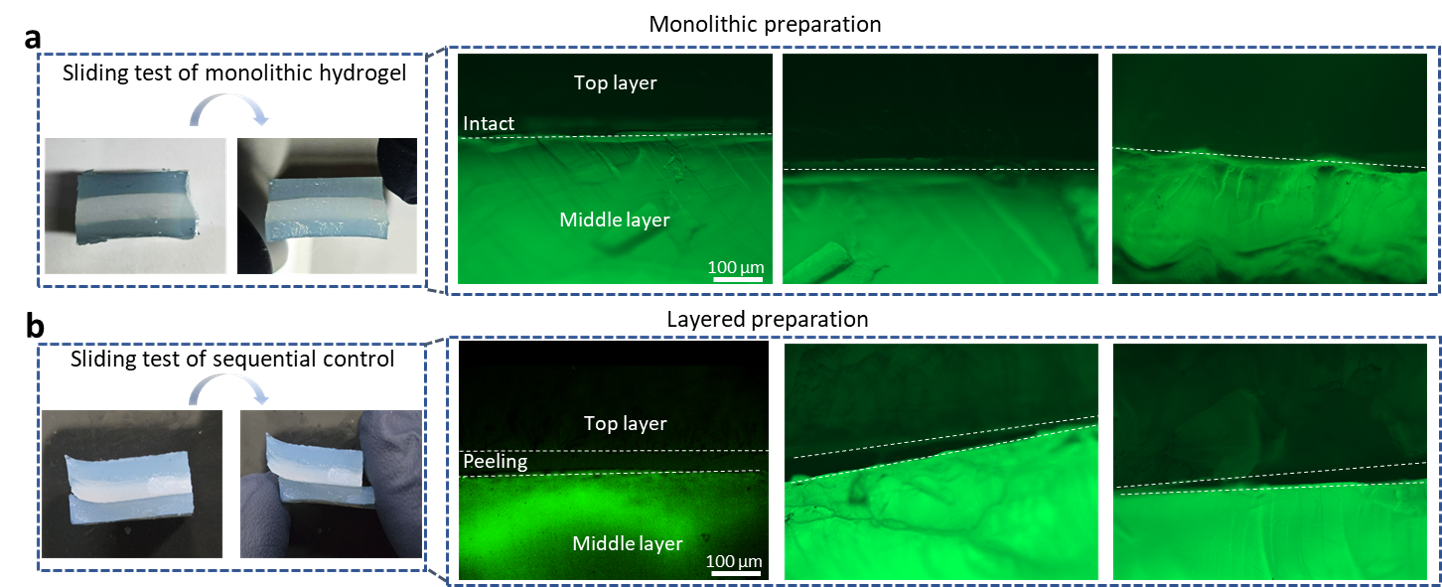

Supplement: Supplementary file 2 — Supporting File 2: advs76893‐sup‐0002‐FigureS1‐S13.zip. [file ADVS-9999-e76893-s002.zip › Figure S5.tif]

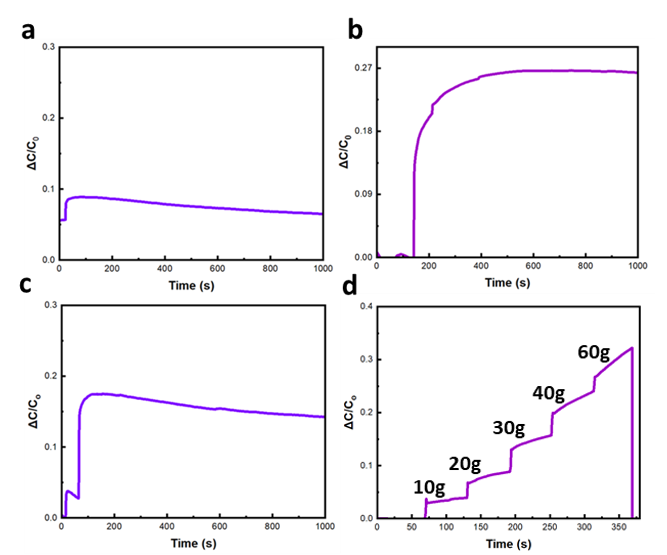

Supplement: Supplementary file 2 — Supporting File 2: advs76893‐sup‐0002‐FigureS1‐S13.zip. [file ADVS-9999-e76893-s002.zip › Figure S6.tif]

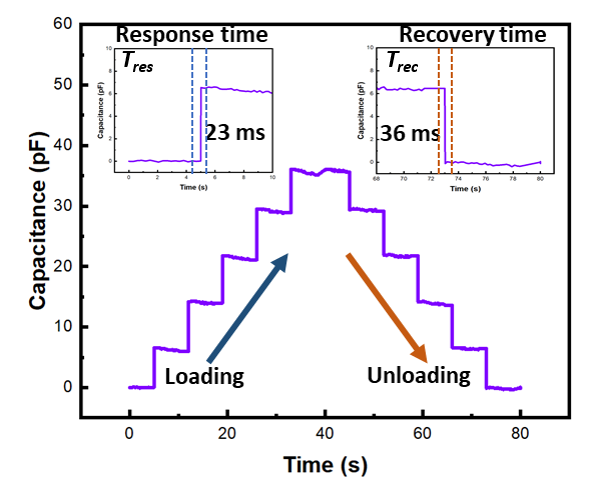

Supplement: Supplementary file 2 — Supporting File 2: advs76893‐sup‐0002‐FigureS1‐S13.zip. [file ADVS-9999-e76893-s002.zip › Figure S7.tif]

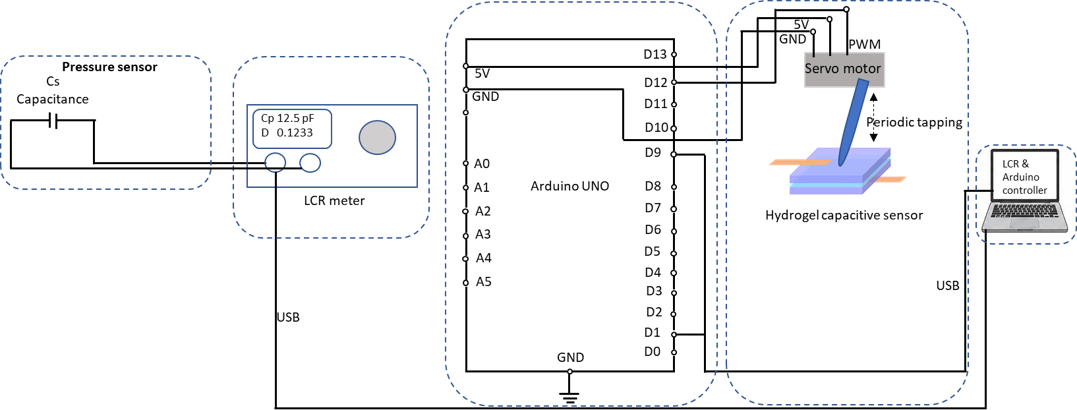

Supplement: Supplementary file 2 — Supporting File 2: advs76893‐sup‐0002‐FigureS1‐S13.zip. [file ADVS-9999-e76893-s002.zip › Figure S8.tif]

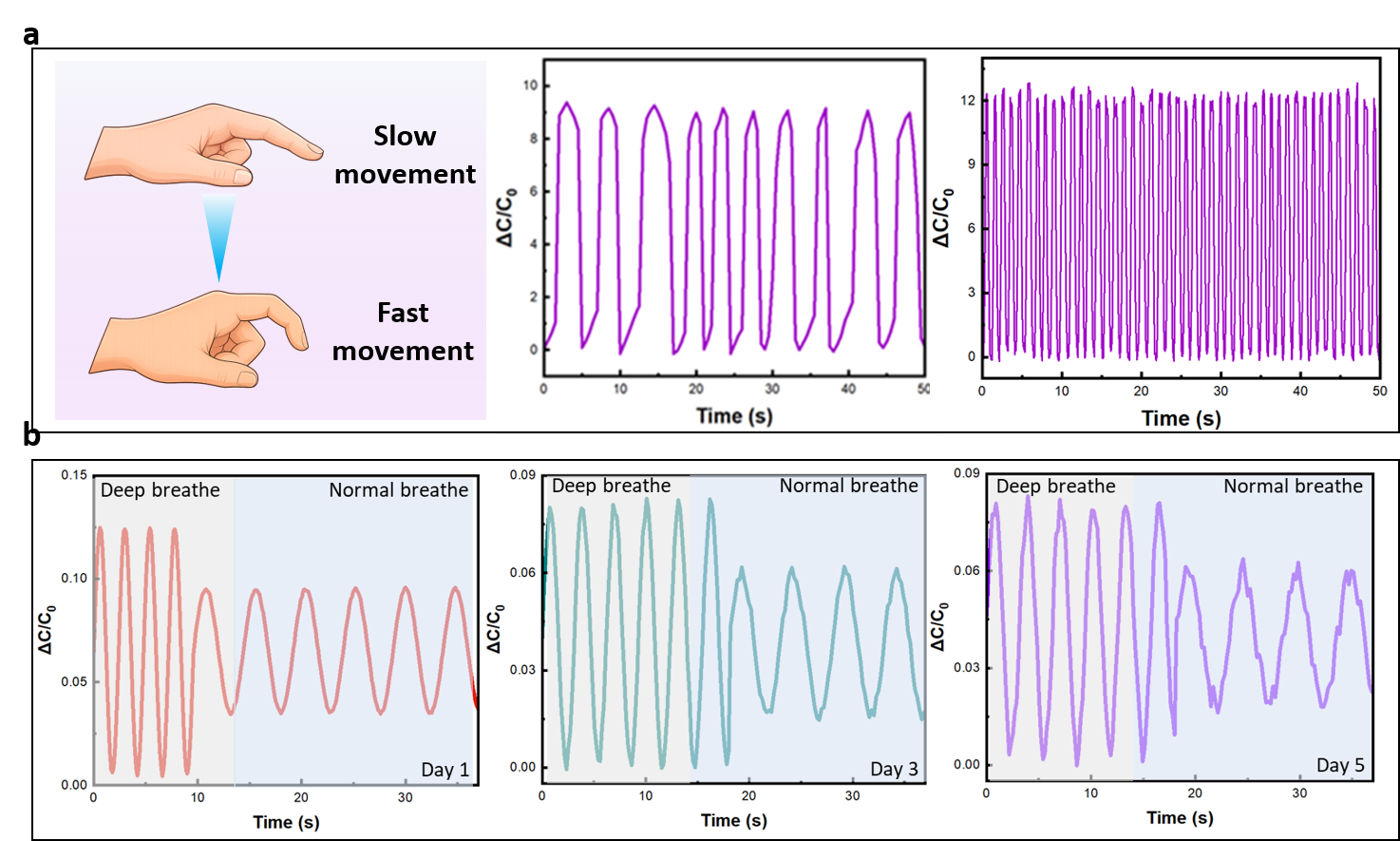

Supplement: Supplementary file 2 — Supporting File 2: advs76893‐sup‐0002‐FigureS1‐S13.zip. [file ADVS-9999-e76893-s002.zip › Figure S9.tif]
